# Controlled human malaria infection with a clone of *Plasmodium vivax* with high-quality genome assembly

**DOI:** 10.1172/jci.insight.152465

**Published:** 2021-12-08

**Authors:** Angela M. Minassian, Yrene Themistocleous, Sarah E. Silk, Jordan R. Barrett, Alison Kemp, Doris Quinkert, Carolyn M. Nielsen, Nick J. Edwards, Thomas A. Rawlinson, Fernando Ramos Lopez, Wanlapa Roobsoong, Katherine J.D. Ellis, Jee-Sun Cho, Eerik Aunin, Thomas D. Otto, Adam J. Reid, Florian A. Bach, Geneviève M.C. Labbé, Ian D. Poulton, Arianna Marini, Marija Zaric, Margaux Mulatier, Raquel Lopez Ramon, Megan Baker, Celia H. Mitton, Jason C. Sousa, Nattawan Rachaphaew, Chalermpon Kumpitak, Nongnuj Maneechai, Chayanut Suansomjit, Tianrat Piteekan, Mimi M. Hou, Baktash Khozoee, Kirsty McHugh, David J. Roberts, Alison M. Lawrie, Andrew M. Blagborough, Fay L. Nugent, Iona J. Taylor, Kimberly J. Johnson, Philip J. Spence, Jetsumon Sattabongkot, Sumi Biswas, Julian C. Rayner, Simon J. Draper

**Affiliations:** 1 The Jenner Institute and; 2 Department of Biochemistry, University of Oxford, Oxford, United Kingdom.; 3 Wellcome Sanger Institute, Wellcome Genome Campus, Cambridge, United Kingdom.; 4 Cambridge Institute for Medical Research, University of Cambridge, Cambridge, United Kingdom.; 5 Mahidol Vivax Research Unit, Faculty of Tropical Medicine, Mahidol University, Bangkok, Thailand.; 6 Institute of Immunology and Infection Research, University of Edinburgh, Edinburgh, United Kingdom.; 7 Walter Reed Army Institute of Research, Silver Spring, Maryland, USA.; 8 Mahidol-Oxford Tropical Medicine Research Unit, Faculty of Tropical Medicine, Mahidol University, Bangkok, Thailand.; 9 Nuffield Division of Clinical Laboratory Sciences, Radcliffe Department of Medicine, University of Oxford, Oxford, United Kingdom.; 10 Department of Pathology, University of Cambridge, Cambridge, United Kingdom.

**Keywords:** Infectious disease, Malaria

## Abstract

Controlled human malaria infection (CHMI) provides a highly informative means to investigate host-pathogen interactions and enable in vivo proof-of-concept efficacy testing of new drugs and vaccines. However, unlike *Plasmodium falciparum*, well-characterized *P*. *vivax* parasites that are safe and suitable for use in modern CHMI models are limited. Here, 2 healthy malaria-naive United Kingdom adults with universal donor blood group were safely infected with a clone of *P*. *vivax* from Thailand by mosquito-bite CHMI. Parasitemia developed in both volunteers, and prior to treatment, each volunteer donated blood to produce a cryopreserved stabilate of infected RBCs. Following stringent safety screening, the parasite stabilate from one of these donors (PvW1) was thawed and used to inoculate 6 healthy malaria-naive United Kingdom adults by blood-stage CHMI, at 3 different dilutions. Parasitemia developed in all volunteers, who were then successfully drug treated. PvW1 parasite DNA was isolated and sequenced to produce a high-quality genome assembly by using a hybrid assembly method. We analyzed leading vaccine candidate antigens and multigene families, including the vivax interspersed repeat (VIR) genes, of which we identified 1145 in the PvW1 genome. Our genomic analysis will guide future assessment of candidate vaccines and drugs, as well as experimental medicine studies.

## Introduction

The majority of human malaria is caused by 2 species of parasite — *Plasmodium falciparum* and *P*. *vivax*. Infection is initiated by an infected *Anopheles* mosquito bite, delivering sporozoites that rapidly migrate to and infect the liver. Asexual replication in the liver sees each infected cell produce thousands of merozoites. These rupture out into the blood and infect RBCs, before undergoing exponential growth that leads to clinical symptoms and the associated morbidity and mortality. *P*. *vivax* is the predominant cause of malaria outside of Africa and is more geographically widespread than *P*. *falciparum*, with 2.5 billion people living at risk in Latin America, Oceania, Asia, and the horn of Africa (1). Moreover, recent data demonstrate a significant burden of morbidity and associated mortality in young children and pregnant women (2), challenging the long-held dogma that this parasite is “benign” (3).

A number of factors also underlie the differing epidemiology of *P*. *vivax* and make it more difficult to control and eliminate than *P*. *falciparum* (4). Most notably, earlier development of gametocytes leads to transmission prior to symptom onset, and its ability to form dormant liver-stage forms, termed hypnozoites, causes waves of relapsing blood-stage parasitemia and sustained transmission (5). However, despite a clear global health need to develop an effective vaccine and improved antimalarial drugs, these efforts continue to lag behind those for *P*. *falciparum*. The reasons for this are numerous, but perhaps most significant is the fact that *P*. *vivax* has not been able to be adapted to long-term in vitro culture, despite extensive efforts. This has severely limited laboratory studies, as well as the development of modern controlled human malaria infection (CHMI) models, which rely on a well-defined isolate of *P*. *vivax* and would enable in vivo efficacy testing of candidate vaccines and antimalarial drugs in proof-of-concept clinical trials. This is in contrast to *P*. *falciparum*, where in vitro culture and sophisticated genetic modification experiments are carried out all over the world; furthermore, CHMI can be initiated by the traditional mosquito-bite method, as well as by injection of cryopreserved sporozoites or an inoculum of blood-stage parasites (6). Most of these studies have been carried out in nonendemic settings, but CHMI trial capacity is now expanding across endemic countries in Africa, enabled by the use of cryopreserved sporozoites. In contrast, modern CHMI with *P*. *vivax* has been less utilized, with only a handful of studies reported (7).

For mosquito-bite *P*. *vivax* CHMI trials, most have taken place in Cali, Colombia (8–11), plus one at the Walter Reed Army Institute of Research (WRAIR; Silver Spring, Maryland, USA) (12), with 108 volunteers challenged in total. Such trials necessitate production of infected mosquitoes in an endemic setting using fresh gametocytes from an infected patient. Shipment of the mosquitoes to nonendemic areas, and timing these activities with recruitment of volunteers who may receive an intervention such as a vaccine, poses significant logistical challenges. Moreover, a different isolate of *P*. *vivax* is inevitably used for every trial, which can hamper interpretation of the results and interstudy comparability. These studies also pose the risk of relapse and, thus, require participants to be screened for glucose-6-phosphate dehydrogenase (G6PD) deficiency (to avoid hemolysis induced by primaquine treatment). They also require assessment of the volunteers’ ability to metabolize primaquine, given that relapsing infection occurred in 2 volunteers in the CHMI study at WRAIR despite primaquine treatment. Here, drug failure was subsequently linked to the volunteers’ cytochrome P450 2D6 (CYP2D6) genotypes that were predicted to be poor or intermediate metabolizer phenotypes of the drug (13).

The use of the blood-stage CHMI model (14, 15) has several advantages over mosquito-bite CHMI, although it does not mimic the route of natural infection. Here, a cryopreserved stabilate of infected RBC (iRBC) is produced from a donor volunteer, enabling subsequent direct blood-stage inoculation of other volunteers with small numbers of parasites. This model is more practical in nonendemic settings; enables access to the parasite’s genetic data before CHMI; removes all risk of relapsing infection; and enables multiple studies with the same strain of parasite (for which a safety database can be established). In the case of *P*. *falciparum*, this model has also proved particularly suitable for estimating the blood-stage parasite multiplication rate (PMR) (16) and for enabling experimental transmission to mosquitoes (17), as compared with studies initiated by mosquito bite. The blood-stage model is also advantageous because it extends the period of blood-stage infection, allowing for longer studies of the human immune response and also allowing for the switching/selection of parasite variant surface antigens (18).

Two cryopreserved stabilates of blood-stage *P*. *vivax* have been reported to date, both produced by the group at the QIMR Berghofer Medical Research Institute (Brisbane, Australia) and obtained from returning travelers who donated infected blood prior to treatment. The first isolate, HMPBS-*Pv* from the Solomon Islands, was safely tested by blood-stage CHMI in 8 volunteers (19, 20); however, this necessitated recruitment of individuals with blood group A to match that of the donor. The second *P*. *vivax* isolate, HMP013-*Pv*, was from India and a blood group O–positive donor. This has been tested in healthy adult volunteers and showed successful induction of gametocytemia and experimental transmission of *P*. *vivax* from humans to mosquitoes (21); it also enabled trials of candidate drugs and further methodology development (22, 23).

Here, we have taken a significant step forward for *P*. *vivax* CHMI by establishing a well-characterized Thai clone of *P*. *vivax* suitable for both mosquito-bite and blood-stage CHMI. We elected to produce a cryopreserved stabilate of iRBC from blood donated by healthy volunteers infected via mosquito-bite CHMI, as opposed to using a blood donation from a returning traveler. This provided numerous advantages in terms of logistical timing and our ability to recruit, in advance, volunteers who passed a full health screen and who had universal donor blood group. In real time, we were able to select mosquitoes infected in Thailand with a single *P*. *vivax* genotype, thus avoiding production of a cryopreserved iRBC stabilate from a polyclonal infection. It also minimized the time from mosquito to blood bank (compared with infected returning travelers); this is important, as it has previously been shown that mosquitoes reset parasite virulence and expression of variant surface antigens (24). Following production of the cryopreserved parasite stabilate, which we called PvW1, we demonstrated safety and infectivity by blood-stage CHMI in 6 healthy adults, and we also report a full genomic analysis of the new PvW1 clone.

## Results

### Source patient case finding and preparation of infected mosquitoes.

For infection of mosquitoes, source patients were recruited from a medical clinic in southern Thailand. Patient blood samples that tested positive for *P*. *vivax* and negative for filarial disease were fed to *Anopheles dirus* mosquitoes via a direct membrane feeding system in Thailand. Oocyst and sporozoite counts subsequently confirmed successful production of 3 independent batches of infected mosquitoes (Supplemental Figure 1A; supplemental material available online with this article; https://doi.org/10.1172/jci.insight.152465DS1). In parallel, and in real time, source patient samples underwent additional and rigorous testing in the United Kingdom for blood-borne infections and mosquito-borne diseases other than malaria; all tests were negative. Nested PCR reported monoinfection with *P*. *vivax* (Supplemental Figure 1, B and C), thus confirming the diagnosis in Thailand; however, genotyping analysis suggested that only 1 blood sample (C05-001) contained a single *P*. *vivax* genotype (Supplemental Figure 1A). Mosquitoes fed off this patient’s blood were, therefore, selected and shipped from Thailand to the United Kingdom.

### Screening of healthy United Kingdom volunteers for blood donation.

In parallel, we enrolled 2 healthy United Kingdom adult volunteers into the VAC068 clinical trial (Supplemental Figure 2). These volunteers were specifically screened to be universal blood donors (blood group O rhesus–negative), Duffy blood group–positive (7, 25), and G6PD-normal (26), and they were screened to have a CYP2D6 genotype predicted to be an extensive metabolizer phenotype (27) alongside satisfactory demonstration of primaquine metabolism following administration of a single test dose of drug (13) (Supplemental Table 1 and Supplemental Figure 3). Each volunteer also underwent an extensive screen for blood-borne infections; all test results were negative; however, both participants were IgG seropositive for Epstein-Barr virus (EBV) and cytomegalovirus (CMV) (Supplemental Table 1), indicating past infection. We did not exclude volunteers based on their serostatus for these 2 viruses.

### Safety and parasite growth dynamics of mosquito-bite CHMI.

For the C05-001 mosquito batch, the mean number of oocysts per mosquito was 3 (range, 0–6) at day 7 after feeding, and the median score for number of sporozoites observed in the salivary glands at day 14 after feeding was +2 (defined as > 10–100 sporozoites) (Supplemental Figure 1A). This was relatively low but sufficient for human transmission. Subsequently, the 2 healthy United Kingdom adult volunteers screened and consented to take part in VAC068 were each exposed to 5 “infectious bites” as defined post–skin feeding by microscopic examination of each mosquito. To achieve this, volunteers 01-004 and 01-008 required 17 and 33 mosquitoes, respectively, to bite their arms.

**Figure 1:**
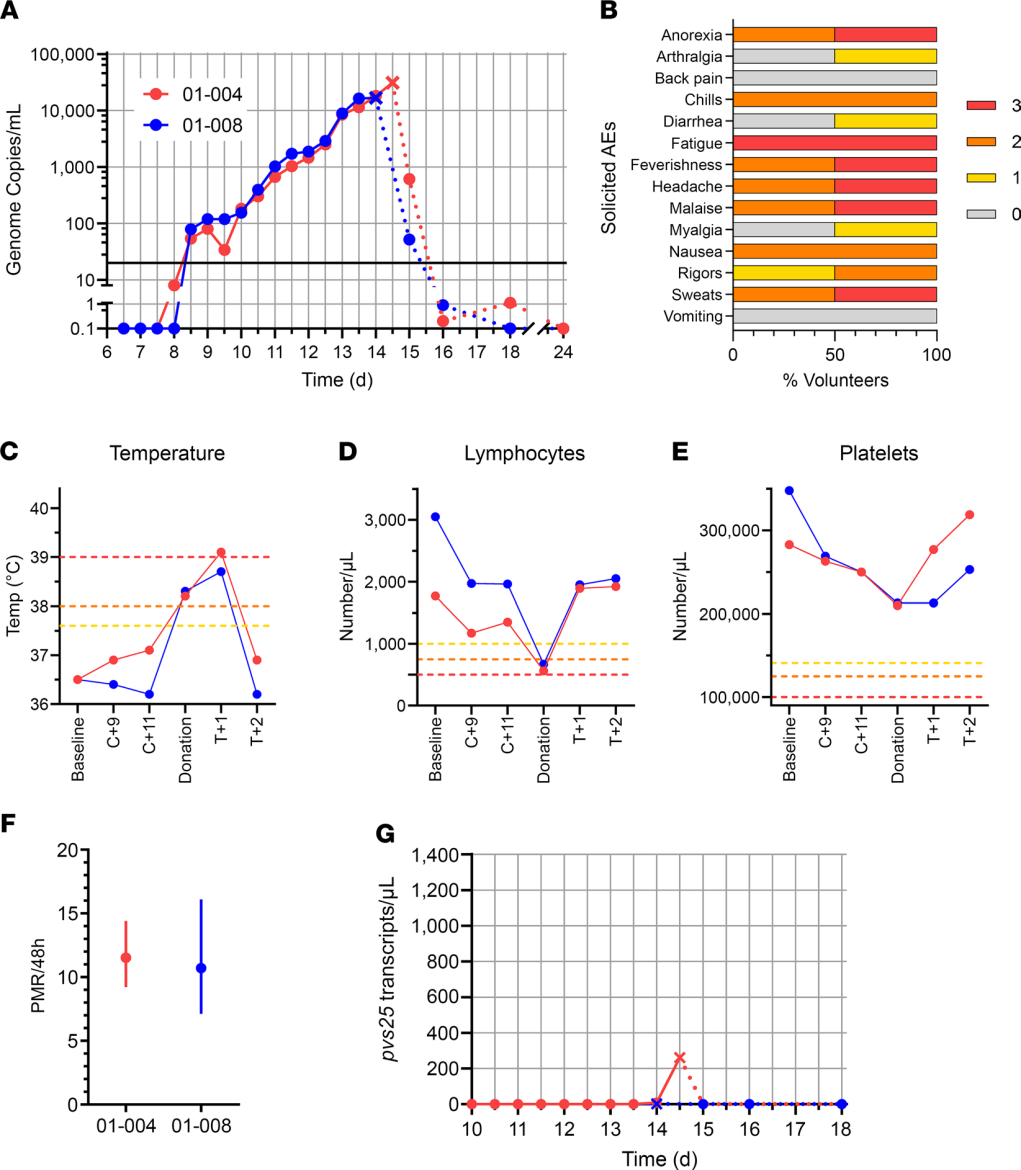
Safety and parasite growth dynamics of *P*. *vivax* sporozoite CHMI. (**A**) qPCR data for the VAC068 trial (*n* = 2). Parasitemia measured in genome copies/mL is shown over time for each volunteer. CHMI was initiated by mosquito bite on day 0. Cross symbols indicate the time point of blood donation followed by antimalarial treatment. Solid lines show qPCR readouts before treatment, and dotted lines after treatment. Solid black line indicates 20 gc/mL (the minimum level to meet positive reporting criteria); samples below this are shown for information only. (**B**) The solicited systemic adverse events (AEs) recorded during the CHMI period (from 1 day up until 45 days after challenge) are shown as the maximum severity reported by each volunteer and as a percentage of the volunteers reporting each individual AE (*n* = 2). Color-coding refers to AE grading: 0 = none; 1 = mild; 2 = moderate; 3 = severe. (**C**) Volunteer temperature (maximum self-recorded by volunteer or measured in clinic) at the indicated time points: baseline before CHMI; 9 and 11 days after CHMI (C+9, C+11); time of blood donation; and 1 and 2 days after treatment (T+1, T+2). AE grading cut-offs are indicated by the dotted lines (yellow = grade 1; orange = grade 2; red = grade 3). (**D** and **E**) Lymphocyte and platelet counts plotted as for **C**. (**F**) The PMR per 48 hours was modeled from the qPCR data up until the time point of blood donation/treatment; PMR ± 95% CI is shown for each volunteer. (**G**) Gametocytemia was assessed over time by qPCR for *pvs25* transcripts; symbols and lines as per **A**.

Parasites were first reliably detected in the blood of both volunteers by quantitative PCR (qPCR) at the evening clinic visit 8 days after CHMI (dC+8.5), and parasitemia then steadily rose over time (Figure 1A and Supplemental Table 2). Over the course of the CHMI period, the 2 volunteers experienced a range of solicited adverse events (AEs), with both reporting grade 3 fatigue and at least grade 2 anorexia, chills, feverishness, headache, malaise, nausea, and sweats (Figure 1B). Both volunteers were admitted for blood donation when they met protocol-specified criteria defined by symptoms and or threshold levels of parasitemia as measured in genome copies (gc)/mL by qPCR. This occurred on the morning of dC+14 for both volunteers, who both crossed the 10,000 gc/mL threshold on dC+13.5 and developed fever on dC+14. Following admission to the clinical trials unit, a 250 mL blood sample was collected (at dC+14 for volunteer 01-008 and dC+14.5 for volunteer 01-004); both were positive by thick film microscopy, and 01-008 and 01-004 reported 16,717 or 31,010 gc/mL by qPCR, respectively. Prior to cryopreservation, these blood samples were then randomized and relabeled either “Donor 1” or “Donor 2” and are now referred to as such in the Results.

After blood donation, each volunteer was immediately treated with Riamet, followed by a 14-day course of primaquine; no supportive treatment or hospital admission was required for either volunteer. Monitoring by qPCR on days 1, 2, 4, 10, and 16 after treatment showed a rapid decline in blood-stage parasitemia, followed by negative readings for both volunteers (Figure 1A and Supplemental Table 2). Most solicited symptoms increased in severity in the first 24 hours after starting antimalarial treatment (Supplemental Figure 4A). Objective fever also increased in the 24 hours after treatment (Figure 1C), and 1 volunteer developed a grade 3 pyrexia (Supplemental Figure 4B); however, all symptoms had completely resolved within 5 days of starting treatment. Both volunteers also experienced some short-lived grade 1 or 2 AEs, possibly related to the antimalarial treatment (dizziness, insomnia, and abdominal pain) (Supplemental Figure 4C). Very few unsolicited AEs (at least possibly related to CHMI) were reported by either volunteer (Supplemental Table 3A), and only 1 grade 3 unsolicited AE (migraine, not related to CHMI) was reported by 01-004 more than 2 months after challenge, requiring attendance to their doctor and resolving within 48 hours (Supplemental Table 3B). Lymphocyte and platelet counts dropped in both volunteers around the time of blood donation (platelets remained within the normal range, but both developed grade 2 lymphocytopenia), rising back to prechallenge levels within 48 hours (Figure 1, D and E, and Supplemental Table 3C). Volunteer 01-008 also developed a transient grade 1 anemia ~6 weeks after challenge (123 g/L at dC+47), which may or may not have been related to CHMI, but this resolved within 3 months (131 g/L at dC+94).

Following completion of the study, the PMR for both volunteers was calculated using a linear model fitted to log_10_-transformed qPCR data (28). These data show comparable PMRs in both volunteers, with 10.7- and 11.5-fold growth per 48 hours (Figure 1F). We also analyzed gametocytemia using a qPCR assay to detect mature female gametocyte *pvs25* transcripts. Volunteer 01-004 showed only low levels at the final time point pretreatment (dC+14.5), while none were detected in volunteer 01-008 (Figure 1G).

Finally, with regard to longer-term safety monitoring, clinic visits at dC+45 and dC+90 gave rise to no safety concerns or indication of relapsing infection, and repeat serological tests for blood-borne infections at dC+90 all remained negative. Ongoing annual follow-up by email will continue for 5 years after CHMI; however, as of the time of writing (3 years after primaquine treatment), no relapse of *P*. *vivax* has been diagnosed for either volunteer (Supplemental Methods).

### Cryopreservation and in vitro testing of P. vivax–infected blood.

**Figure 2:**
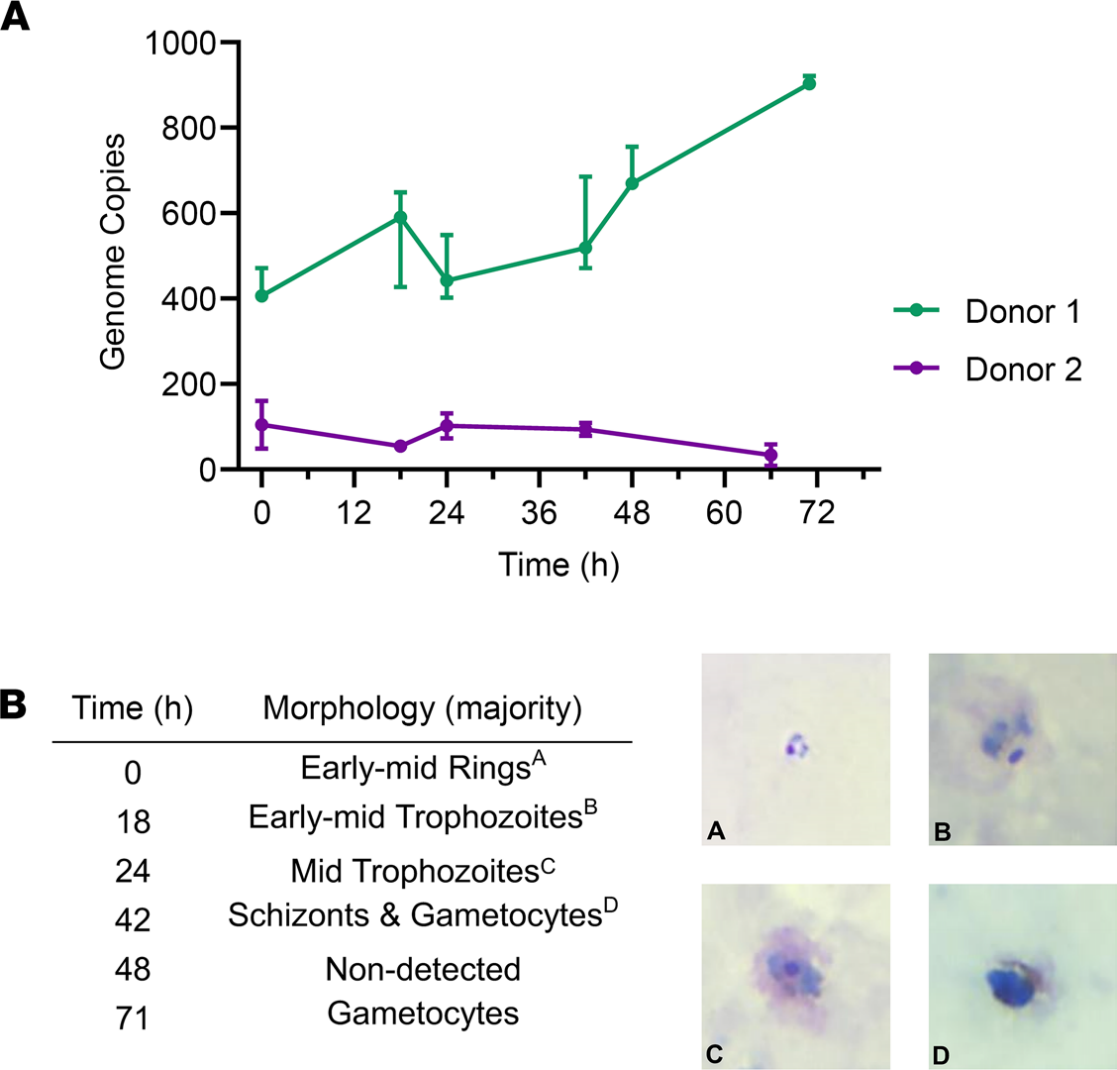
Test of cryopreserved parasite viability by short-term in vitro culture assay. (**A**) Test vials of cryopreserved parasites from Donor 1 and Donor 2 were thawed, and cells were used in a short-term in vitro parasite culture assay. *P*. *vivax* parasite growth was monitored by qPCR in 20 μL samples of RBC extracted at the indicated time points. Median and range of triplicate readings are shown in genome copies measured per 20 μL sample. (**B**) Parasite morphology was monitored at the same time points over the first growth cycle by light microscopy of Giemsa-stained thick and thin blood films Representative images are shown from Donor 1, and the predominant morphology observed is reported. Total original magnification, ×1000.

After blood donation, the leukodepleted blood from both volunteers in VAC068 was processed, and the RBCs were mixed with Glycerolyte 57 to form a stabilate prior to cryopreservation. In total, 190 vials were frozen for Donor 1, and 185 were frozen for Donor 2. Testing by qPCR indicated minimal or no loss of parasites during filtration (95% and 105% recovery for Donor 1 and Donor 2, respectively). We next tested for parasite viability in both cryopreserved stabilates. Vials were thawed, and cells were used in a short-term in vitro parasite culture assay, since *P*. *vivax* cannot currently be cultured long-term in vitro. Parasite growth was detectable by qPCR and light microscopy through 1 initial growth cycle in samples collected from Donor 1, with normal progression of parasite morphology seen on Giemsa-stained thick and thin blood films (Figure 2). However, no growth was discernible in samples obtained from Donor 2. We therefore undertook further quality control testing on vials from Donor 1, with the material tested for sterility, mycoplasma, and endotoxin; the materials passed all tests. Another screen for blood-borne infections was also conducted on the plasma derived directly from the blood donation; all tests were negative.

Finally, we also screened Donor 1 for the Kell blood group antigen because women of childbearing potential who receive a blood transfusion have a small additional risk of developing RBC alloantibodies that could cause problems during pregnancy. In particular, there is a potential risk of development of hemolytic disease of the newborn in relation to Kell antigen incompatibility — i.e., if Kell-positive donor blood is transfused to a Kell-negative female recipient. However, testing of the donor’s blood sample confirmed Kell antigen negativity, thereby allowing future universal administration of the cryopreserved *P*. *vivax* iRBC stabilate with respect to sex.

### PvW1 infectivity, parasite growth dynamics and safety of blood-stage CHMI.

Given that all safety and viability tests were passed for the cryopreserved stabilate of *P*. *vivax* iRBC from Donor 1, we named this clonal isolate “PvW1” and proceeded to test safety and infectivity by blood-stage CHMI. We therefore recruited 6 healthy, malaria-naive United Kingdom adults into the VAC069A clinical trial, comprising 3 groups of 2 volunteers (Supplemental Figure 5), and we tested feasibility of infection at 3 different doses of PvW1 blood-stage inoculum. Five vials of the PvW1 cryopreserved stabilate were thawed and then combined to produce a single batch of blood-stage inoculum. Two volunteers received a whole vial’s worth of iRBC (“neat”), 2 volunteers received a 20% challenge dose via a 1:5 dilution, and the final 2 volunteers were inoculated with a 5% dose via a 1:20 dilution. All 6 volunteers underwent blood-stage CHMI at the same time.

**Figure 3:**
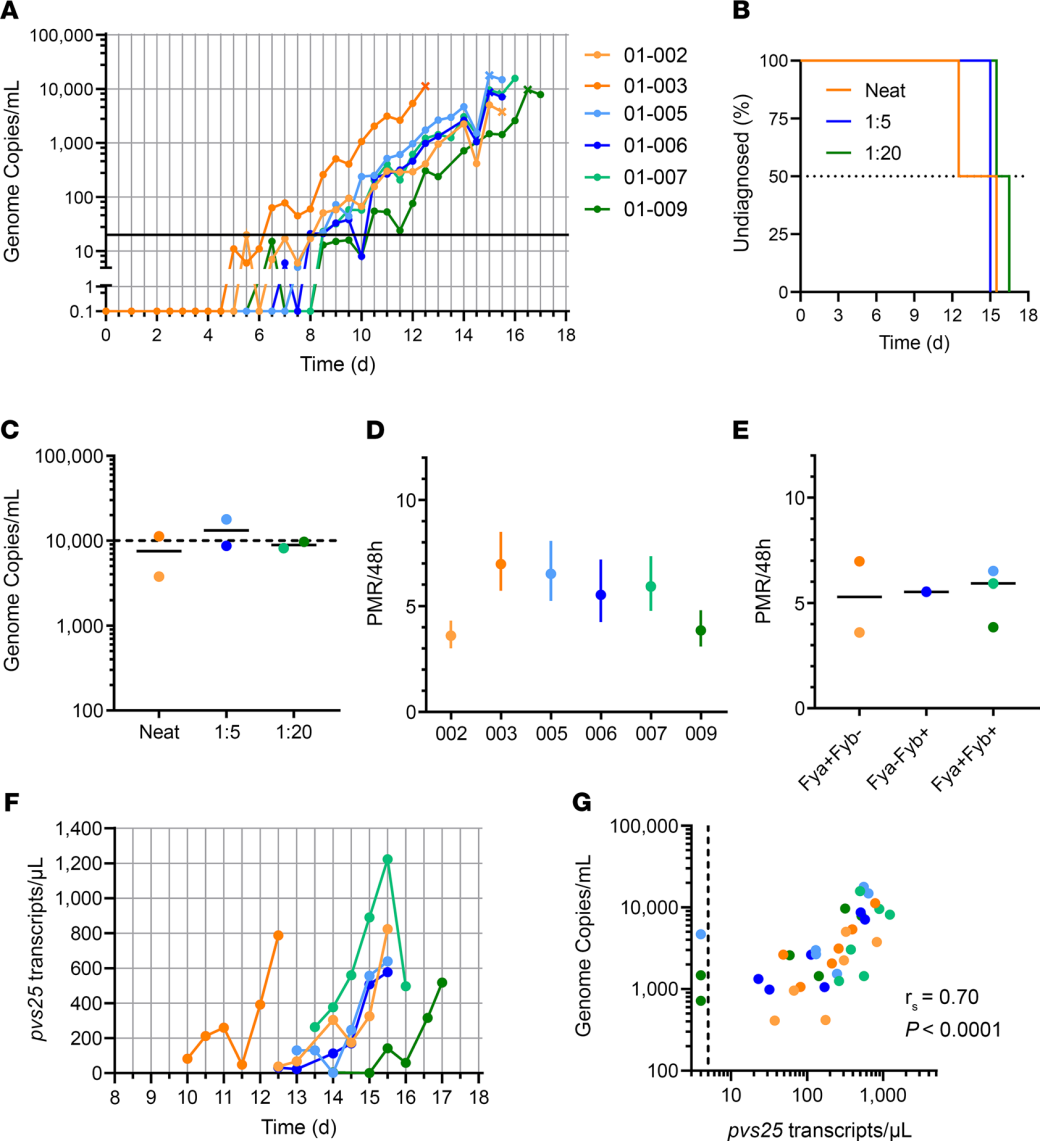
Parasite growth dynamics of *P*. *vivax* PvW1 clone blood-stage CHMI. (**A**) qPCR data for the VAC069A trial (*n* = 6). Parasitemia measured in genome copies (gc)/mL is shown over time for each volunteer. CHMI was initiated by blood-stage inoculation on day 0. Cross symbols indicate the time point of diagnosis. Orange = neat inoculum dose; blue = 1:5; green = 1:20 dilution of the neat inoculum dose. Solid black line indicates 20 gc/mL (the minimum level to meet positive reporting criteria); samples below this are shown for information only. (**B**) Kaplan-Meier plot of time to diagnosis in days for the VAC069A study (*n* = 2/group). (**C**) Parasitemia measured in gc/mL at the time point of diagnosis. Individual data points and median are indicated for each dose group. Volunteers were diagnosed when they reached a threshold of 10,000 gc/mL OR if they had symptoms of malaria with a parasitemia > 5,000 gc/mL. (**D**) The PMR per 48 hours was modeled from the qPCR data up until the time point of diagnosis; PMR ± 95% CI is shown for each volunteer. (**E**) Individual and median PMR are shown with volunteers grouped according to their Duffy blood group antigen (Fy) serological phenotype. (**F**) Gametocytemia was assessed over time by qPCR for *pvs25* transcripts; colored lines as per **A**. (**G**) Correlation of total parasitemia measured in gc/mL versus *pvs25* transcripts/μL. Spearman’s rank correlation coefficient and *P* value are shown; *n* = 36.

Blood-stage parasitemia was monitored as previously by qPCR, beginning 1 day after challenge (dC+1) (Figure 3A and Supplemental Table 4). All 6 volunteers were successfully infected, with a median time to diagnosis of 15.25 days after CHMI (range, 12.5–16.5) (Figure 3B). The median parasitemia at diagnosis across all 6 volunteers was 9178 (range 3779–17,795) gc/mL (Figure 3C). We also calculated the PMR as before using a linear model fitted to log_10_-transformed qPCR data (28). These data show a median of 5.7-fold (range, 3.6-fold to 7.0-fold) growth per 48 hours across the 6 volunteers (Figure 3D), notably lower than that previously observed in the mosquito-bite CHMI study (Figure 1F). There was also no discernible difference in the PMRs across the 3 different challenge dose cohorts (Figure 3D) or across the 3 different Duffy blood group serophenotypes, all with median values between 5.3- and 5.9-fold growth per 48 hours (Figure 3E). We also analyzed gametocytemia at the 6 time points preceding diagnosis for each volunteer, and we observed rising levels in all individuals (Figure 3F). This was in clear contrast to the observations after mosquito-bite CHMI (Figure 1G) and despite comparable (if not slightly lower) levels of overall blood-stage parasitemia as measured in gc/mL. Here, we also saw a strong positive correlation between the measured overall levels of parasitemia in gc/mL versus *pvs25* transcripts/μL (Figure 3G).

**Figure 4:**
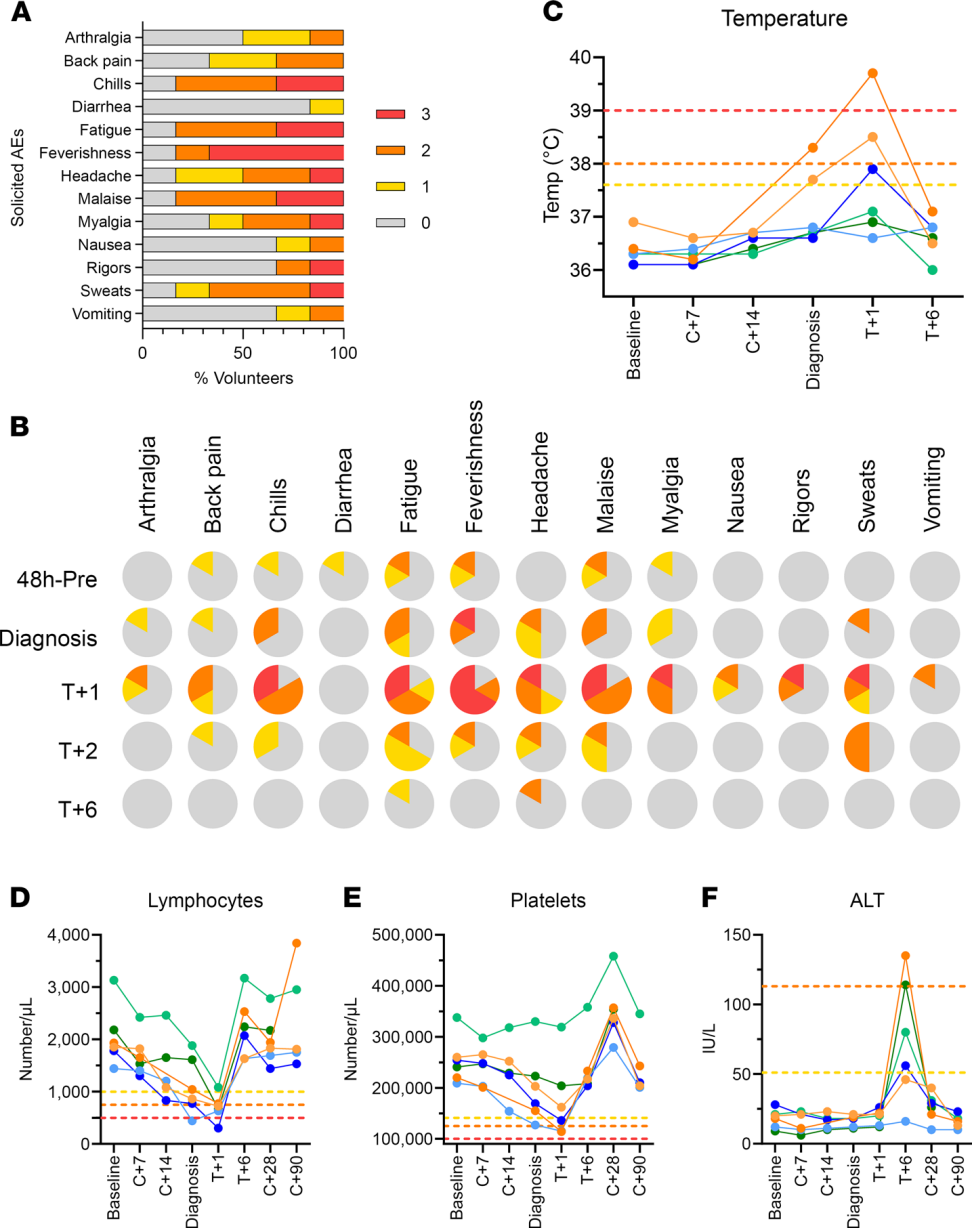
Safety analysis of *P*. *vivax* PvW1 clone blood-stage CHMI. (**A**) The solicited systemic adverse events (AEs) recorded during the CHMI period (from 1 day up until 90 days after challenge) are shown as the maximum severity reported by each volunteer and as a percentage of the volunteers reporting each individual AE (*n* = 6). Color-coding refers to AE grading: 0 = none; 1 = mild; 2 = moderate; 3 = severe. (**B**) The solicited systemic AEs recorded at the indicated time points during the CHMI period are shown as the maximum severity reported by each volunteer and as a percentage of the volunteers reporting each individual AE (*n* = 6). Color-coding as per **A**. 48h-pre = the 48 hour period prior to *P*. *vivax* diagnosis; Diagnosis = time point of diagnosis; T+1, T+2, and T+6 = indicated days after treatment. (**C**) Volunteer temperature (maximum self-recorded by volunteer or measured in clinic) at the indicated time points: baseline before CHMI; 7 and 14 days after CHMI (C+7, C+14); time of diagnosis; and 1 and 6 days after treatment (T+1, T+6). AE grading cut-offs are indicated by the dotted lines (yellow = grade 1; orange = grade 2; red = grade 3). (**D**–**F**) Lymphocyte (**D**) and platelet counts (**E**), and alanine aminotransferase (ALT) measurements (**F**), all plotted as for **C** but also including C+28 and C+90 time points.

With regard to safety, there were no serious AEs (SAEs) in the VAC069A study, and all volunteers completed treatment without complication. One volunteer withdrew at dC+28, with the remaining 5 completing clinical follow-up at dC+90 (Supplemental Figure 5). The maximum severity of solicited AEs at any time during the CHMI period is shown for all 6 volunteers in Figure 4A, with 4 volunteers reporting grade 3 solicited AEs (most commonly feverishness) persisting for 24 hours and 1 for 48 hours (Supplemental Table 5A). The proportion of volunteers reporting solicited AEs — specifically prediagnosis, peridiagnosis, and posttreatment AEs — is shown in Figure 4B. Around the time of diagnosis, 33%–50% of the volunteers reported mild-to-moderate symptoms — mainly fatigue, headache, myalgia, malaise, feverishness, and chills. Symptoms peaked in severity in the first 24 hours after starting antimalarial treatment with Riamet or Malarone, with only 1 volunteer remaining asymptomatic (Figure 4B). Objective fever also increased in the 24 hours after treatment, with 3 of 6 volunteers developing pyrexia (1 of each grades 1–3; Figure 4C and Supplemental Figure 6A). Nevertheless, most symptoms had completely resolved within a few days of starting treatment, and only 1 volunteer still had headache and fatigue at 6 days after starting treatment (T+6) (Figure 4B). Three volunteers (50%) also experienced short-lived AEs, possibly related to the antimalarial drugs (50% moderate dizziness; 33% mild insomnia, cough, and palpitations) (Supplemental Figure 6B). Very few unsolicited AEs (at least possibly related to CHMI) were reported by any of the volunteers (Supplemental Table 5B).

With regard to laboratory AEs (Supplemental Table 5C), lymphocyte counts dropped significantly in 4 of 6 volunteers around the time of diagnosis or 1 day after treatment (grade 3 lymphocytopenia in 2 volunteers), but all counts normalized within 6 days of starting treatment (Figure 4D). Two volunteers developed a short-lived grade 2 thrombocytopenia, again normalizing within 6 days of treatment (Figure 4E); however, 2 volunteers also developed a mild-moderate anaemia after diagnosis. With regard to the latter, 1 normalized within 28 days of challenge and the other persisted at grade 1 at dC+90 (102 g/L) and was therefore referred to their medical practitioner for ongoing monitoring as a precautionary measure (Supplemental Table 5C and Supplemental Figure 6C). The only notable change in blood chemistry was a transient grade 1–2 rise in the ALT in 4 of 6 volunteers, captured consistently at 6 days after treatment (Figure 4F). All ALT levels fully resolved to prechallenge levels with no associated abnormalities in other indices of liver function (Supplemental Figure 6C and Supplemental Table 5D). Finally, we also confirmed CMV and EBV sero-status of all volunteers before and after CHMI. All 6 volunteers were EBV sero-positive before CHMI, and 3 were CMV sero-positive. Of the 3 CMV sero-negative volunteers, 1 withdrew consent and left the trial at C+28 and was therefore not retested, while the other 2 remained sero-negative when retested at C+90.

### Antibody responses to blood-stage merozoite antigens after CHMI.

**Figure 5:**
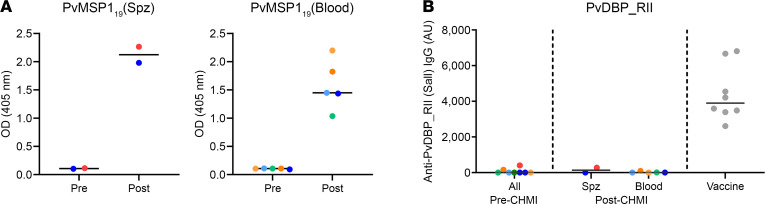
Induction of serum antibody responses to merozoite antigens during CHMI. (**A**) Serum anti-PvMSP1_19_ IgG ELISA was conducted on samples from the VAC068 mosquito-bite/sporozoite (spz) CHMI study (*n* = 2) and the VAC069A blood-stage CHMI study (*n* = 5, because 1 volunteer withdrew at dC+28). OD 405 nm data are shown for sera tested at a 1:100 dilution from the pre-CHMI (dC–1) and 90 days post-CHMI (dC+90) time points. Samples color-coded as per previous figures. (**B**) Serum anti-PvDBP_RII (SalI allele) IgG as measured by standardized ELISA, reporting in arbitrary units (AU). Same samples tested as in **A**. Vaccine = positive control samples (*n* = 8) from a previous Phase Ia clinical trial of a PvDBP_RII vaccine (29). Individual data points and median are shown.

We next assessed for the induction of serum IgG antibody responses after CHMI against 2 well-known blood-stage merozoite antigens — *P*. *vivax* merozoite surface protein 1 C-terminal 19 kDa region (PvMSP1_19_) and *P*. *vivax* Duffy-binding protein region II (PvDBP_RII). All volunteers had detectable IgG against PvMSP1_19_ after CHMI, with similar results seen in the VAC068 mosquito-bite sporozoite CHMI study and the VAC069A blood-stage CHMI study (Figure 5A). However, there were no detectable responses after CHMI against PvDBP_RII in any of the volunteers, in contrast to positive control samples from a cohort of healthy United Kingdom adult volunteers previously vaccinated with the PvDBP_RII antigen (29), which were included here for comparison (Figure 5B). We also assessed for the induction of serum IgG antibody responses after CHMI against the well-known preerythrocytic antigen, *P*. *vivax* circumsporozoite protein (PvCSP). Responses were negative before CHMI in both VAC068 volunteers, with no evidence of seroconversion to PvCSP at dC+90 after sporozoite CHMI (data not shown).

### PvW1 genome assembly allows resolution of complex multigene families.

Finally, we produced a genome assembly for PvW1 by using a hybrid assembly method, which combined long PacBio reads with short Illumina reads. The PvW1 genome assembled into 14 scaffolds (the 14 *P*. *vivax* chromosomes) and is comparable in both assembly size and number of genes to the highest quality existing *P*. *vivax* assembly, PvP01 (30) (Table 1). The PvW1 assembly has fewer unassigned scaffolds than any other assembly, indicating the completeness of the assembled genome and the benefits of using a combination of long and short reads; note that PvP01, PvC01, and PvT01 were all assembled using Illumina data only (30), while the original reference, PvSalvador-1 (SalI), was created using capillary sequence data (31).

**Figure 6:**
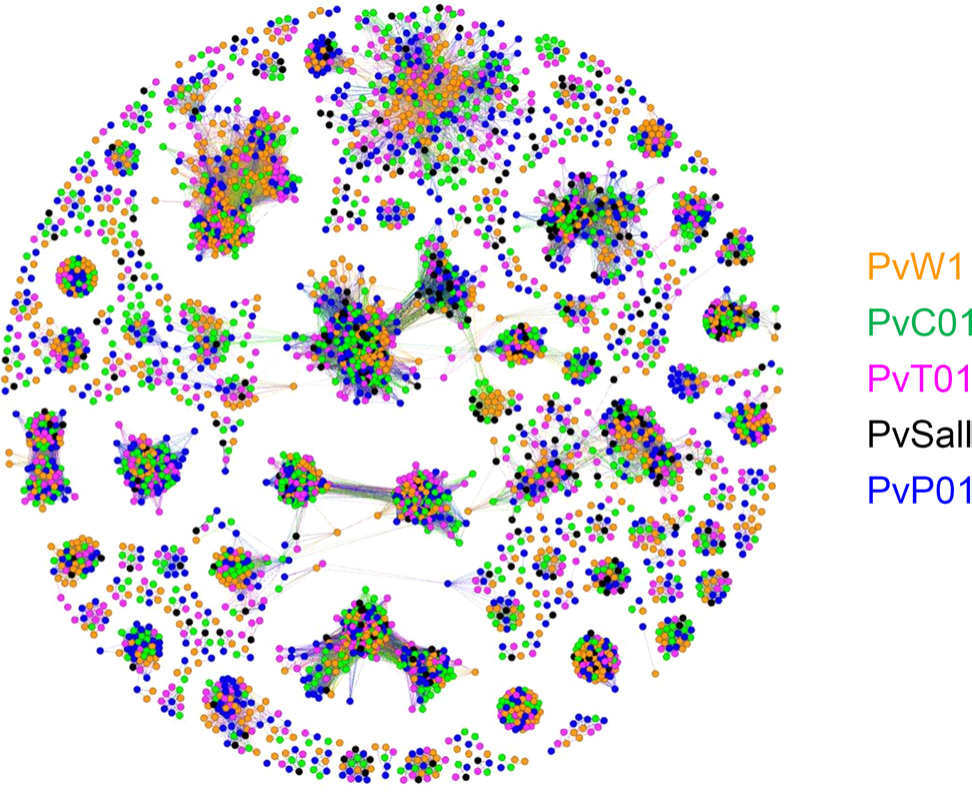
Cluster analysis of the PvW1 VIR proteins. Cluster analysis of the 1145 predicted VIR proteins encoded by the PvW1 genome compared with those of other *P*. *vivax* isolates (30, 31). Each spot represents a VIR protein from either PvW1 (orange), PvC01 (green), PvT01 (pink), PvSalI (black), and PvP01 (blue). Relatedness between the proteins is represented by distance; therefore, more closely related proteins cluster together. Most of the clusters contain proteins from several isolates, suggesting that the clusters are not restricted to specific genomes or geographical distribution.

The high quality of the PvW1 assembly allowed us to identify 1145 vivax interspersed repeat (VIR) genes within the genome, comparable in number to the PvP01 genome. Computational studies have shown that the VIR genes from different *P*. *vivax* isolates can be grouped into a number of clusters, and it is possible that genes within clusters may be performing a similar function (30, 32). Cluster analysis showed that the majority of the 1145 PvW1 VIR proteins could be clustered into groups with VIRs from the PvP01, PvT01, PvC01, and SalI strains (Figure 6), with no evidence that specific clusters are restricted to specific genomes or geographical regions. Of 206 VIR clusters that had > 5 genes and, therefore, had the potential to include VIR representatives from all 5 isolates, 98 were missing at least 1 isolate. However, in 90 of those cases, the missing isolate was SalI. As shown in Table 1, the SalI genome, which was sequenced more than 10 years ago using earlier genome sequencing technology, has significantly fewer VIR genes, presumably because such genes are concentrated in subtelomeric regions that are largely unassembled in that genome. There were only 24 clusters that were missing at least 1 isolate from the more fully assembled genomes (PvW01, PvT01, PvC01, and PvP01); if the size of the cluster was increased to greater than 8 genes, that number of clusters missing at least 1 isolate dropped to 9. This emphasizes that the vast majority of clusters appear to be present across genomes and geographic regions, causing us to make the hypothesis that the clusters may have primarily emerged before the broad geographic dispersal of *P*. *vivax.* Similarly, we resolved other smaller but still highly polymorphic multigene families such as the merozoite surface protein 3 (MSP3) family. These proteins are expressed on the surface of the invasive merozoite and are known to be highly polymorphic both in sequence and gene number between isolates. We compared the organization of the MSP3 multigene family in PvW1 to *P*. *vivax* isolates: PvP01 (30) and SalI, India-7, North Korean, Mauritania-1, and Brazil-1 (33). Genes flanking the MSP3 cluster (PVX_097665 and PVX_097740) are syntenic across all isolates, as are MSP3.1, MSP3.2, MSP3.3, MSP3.G, MSP3.10, and MSP3.11. There is, however, clearly variability in the central region of the MSP3 region, with MSP3.4, MSP3.5, MSP3.6, MSP3.7, MSP3.8, and MSP3.9 all present in some isolates but not others (Supplemental Figure 7). The arrangement of the PvW1 MSP3 cluster appears identical to that of PvP01.

### PvW1 vaccine candidate and drug resistance–associated genes.

The quality of the PvW1 genome also makes it easy to obtain and analyze potential vaccine targets, which we did for 3 high-profile candidates (34), comparing the PvW1 sequence with those from PvP01 and SalI. The sporozoite-stage target PvCSP is known to contain 1 of 2 major types of repeat called VK210 and VK247 (35, 36), and this heterogeneity is an important factor for vaccine design. PvW1 contains VK210 repeats, the most prevalent form worldwide (Supplemental Figure 8A). The sequence of the transmission-stage candidate Pvs25 is highly conserved between PvW1 and other genomes, apart from the commonly variable amino acids 130 and 131 within the third epidermal growth factor–like (EGF-like) domain (Supplemental Figure 8B). Finally, we reviewed the PvDBP sequence, since 2 vaccine candidates targeting region II are currently in early-phase clinical trials (29, 37). PvDBP in PvW1 has multiple polymorphisms with 10 in region II, including the DEK epitope (38), as compared with the SalI sequence used in the current clinical vaccines (29, 37). Like PvDBP from SalI, this gene in PvW1 also has a 9–amino acid deletion (downstream of region II) that is not present in PvP01 (Supplemental Figure 8C). Beyond varying at a sequence level, PvDBP is also known to vary between isolates in copy number, with some isolates containing multiple copies (39, 40) now linked to evasion of humoral immunity (41). We, therefore, used Illumina read mapping across the PvW1 genome assembly to check for copy number variation of genes. Here, if regions of the genome are present in multiple copies, then the read coverage over that region would be higher than the surrounding regions. There was no evidence for increased coverage at either PvDBP or its homologue PvDBP2 (also called *P*. *vivax* erythrocyte-binding protein [PvEBP]), suggesting that both are present at a single copy within the PvW1 genome (Supplemental Figure 9, A and C). We also looked at an uncharacterized gene on chromosome 14, homologous to PVX_101445/PvP01_1468200, which has been shown to be duplicated in some isolates (42). This gene is also present in a single copy in PvW1 (Supplemental Figure 9D).

Drug resistance is not as well characterized in *P*. *vivax* as in *P*. *falciparum,* but several genes and polymorphisms have been associated with resistance in field studies. We, therefore, examined the sequences of 4 genes within the PvW1 genome that have been associated with drug resistance: dihydrofolate reductase (*PvDHFR*), dihydropteroate synthetase (*PvDHPS*), chloroquine resistance transporter (*PvCRT*), and multidrug resistance transporter 1 (*PvMDR1*) (Supplemental Figure 9B). The PvW1 *PvDHFR* gene encodes a protein with the quadruple mutation F57L/S58R/T61M/S117T that has been linked to pyrimethamine resistance (43), whereas *PvDHPS* showed no mutations previously associated with sulfadoxine resistance (44). The molecular basis of *P*. *vivax* chloroquine resistance is less clear, although there is some evidence that mutations in *PvCRT* (K10 insertion) and *PvMDR1* (Y976F mutation) may be involved (45–47). Neither of these mutations are present in the PvW1 *PvCRT* and *PvMDR1* genes. It is important to note that both Riamet (a combination of artemether and lumefantrine) and Malarone (a combination of atovaquone with proguanil) antimalarials were used with 100% treatment success rates in the VAC068 and VAC069A studies (both volunteers in VAC068 and 5 of 6 volunteers in VAC069A received Riamet; 1 of 6 received Malarone), and none of the polymorphisms identified have been associated with resistance to either of these drugs.

## Discussion

Here, we undertook CHMI model development for *P*. *vivax* and established a potentially new PvW1 clonal isolate from Thailand. Our methodology elected to focus on a mosquito-bite CHMI protocol to provide the initial source of blood-stage parasites for the cryopreserved stabilate. The main advantages here (over parasites donated by returning travelers) included the ability to control the parasite source, the recruitment of suitable healthy volunteers (especially with regard to health screening and universal donor blood group), and logistical timing. We also created the blood stabilate as close as possible to the mosquito stage, with only ~3 cycles of replication from the liver (since it is known that mosquitoes reset parasite virulence; ref. 24). If parasites had been cryopreserved from returning travelers or chronically infected adults in an endemic setting, they would have been selected over many rounds of asexual replication in vivo before creating the stabilate. This diminishes the criticism that blood-stage CHMI is not the natural route of infection. Furthermore, as many as 80% of *P*. *vivax* blood-stage infections are caused by relapsing parasites, which means that, in the unique context of relapsing *P*. *vivax*, a challenge with recently emerged blood stages is, in many ways, closer to most “natural challenges” than mosquito bite–delivered sporozoites. That being said, it is important to acknowledge that blood-stage CHMI is only useful to measure interventions against the blood stage of infection rather than sporozoites or hypnozoite establishment.

Notably our real-time assessment of parasite genotypes in the infected mosquitoes in Thailand identified only 1 clonal infection out of 3 tested. In the future, it will likely be necessary to screen more infected patient samples if parasite clones with specific genotypes are desired. It is also probable that this clonal infection resulted from a single relapsing hypnozoite in the patient, since natural infections are frequently polyclonal, arising from primary infections with multiple genotypes and meiotic siblings produced in the mosquito and/or multiple heterologous hypnozoites relapsing at a similar time (48–50).

The VAC068 mosquito-bite trial demonstrated feasibility and safety of this CHMI model for the first time to our knowledge at a European site, albeit in only 2 healthy adult United Kingdom volunteers. Both were successfully infected, with parasites first detectable by qPCR on dC+8.5 and the first wave of blood-stage parasitemia peaking around dC+9. This is largely consistent with data from humanized mouse models suggesting that the complete maturation of *P*. *vivax* liver stages and exoerythrocytic merozoite release occurs between days 9 and 10 after sporozoite infection (51). Growth of blood-stage parasitemia was subsequently similar in the 2 volunteers, with both meeting criteria to donate blood on dC+14, prior to radical cure treatment with Riamet followed by primaquine. Both volunteers were screened to have CYP2D6 genotypes predicted to be extensive metabolizer phenotypes of primaquine, and as of ~3 years of long-term follow-up, no relapse of infection has been documented.

Cryopreservation of the iRBC stabilate was performed successfully; however, given that *P*. *vivax* cannot be cultured long-term in vitro, it proved challenging to confirm parasite viability following thaw of the frozen stabilate, especially given the relatively low level of parasitemia achieved by CHMI in nonimmune adults. However, since the stabilate from Donor 1 showed demonstrable growth in vitro using a short-term culture assay, we elected to proceed with this material for onward testing. Poor parasite recovery from Donor 2 could be associated with the predominant life cycle stage at the time of cryopreservation; here, microscopy records indicate the presence of more schizonts and a smaller proportion of early ring-stage trophozoites in comparison with Donor 1. Previous evidence suggests that the late asexual intraerythrocytic parasites are not viable after cryopreservation with glycerolyte (52), and this may have led to the poor recovery of live parasites in Donor 2’s stabilate.

Previous reports of blood-stage CHMI using *P*. *vivax* have used 1 vial of cryopreserved stabilate to infect 1 volunteer (19–21), in contrast to similar studies with the stabilate of 3D7 clone *P*. *falciparum*, whereby a single vial is diluted and routinely used to infect about 20–30 volunteers (16, 53). Thawing many vials to undertake CHMI in larger cohorts of volunteers — e.g., for vaccine efficacy trials — brings many practical difficulties and, in turn, more rapidly depletes the bank of cryopreserved stabilate, which is a finite resource. Conserving vials and building up a long-term safety database of the challenge agent for future use across many clinical studies is also preferable. Consequently, we assessed 3 different doses of the PvW1 blood-stage inoculum in the VAC069A study, with 2 volunteers receiving each dose. All 6 volunteers were successfully diagnosed at similar levels of blood-stage parasitemia within 12–16 days. Importantly, these data suggest that blood-stage CHMI trials in larger volunteer cohorts are now practical and feasible, while preserving the bank of PvW1 parasites for the long-term.

The AE profiles of both the mosquito-bite and blood-stage CHMI with PvW1 were highly comparable with previous reports of both models in malaria-naive/nonimmune adults using other isolates of *P*. *vivax* at the Colombian (8–10), American (54), or Australian sites (19–23). No SAEs occurred in either trial, and all drug treatments were successful. Symptoms consistent with malaria were experienced and peaked after treatment prior to resolving within a few days. We also observed transient thrombocytopenia and lymphocytopenia, as well as rises in ALT 6 days after treatment, consistent with the reports of other sites undertaking *P*. *vivax* CHMI (9, 10, 55) and with no apparent impact on volunteer safety. We also observed consistent sero-conversion to PvMSP1_19_ after CHMI in all volunteers, as reported in the Colombian CHMI trials (10, 56), but we observed no detectable responses to PvDBP_RII or PvCSP. These data for the merozoite antigens are in line with our similar studies of *P*. *falciparum* CHMI, with sero-conversion of malaria-naive adults observed to immunodominant merozoite surface proteins following primary acute malaria exposure but not to more transiently exposed RBC invasion ligands (57, 58).

Following mosquito-bite CHMI, we observed ~10-fold growth in blood-stage parasitemia per 48 hours, consistent with other reports for *P*. *vivax* (20) and our experience with *P*. *falciparum* (16, 53). Interestingly, however, the average PMR was lower (~5.5-fold growth per 48 hours) following blood-stage CHMI with the same parasite. There was no obvious effect of challenge dose or Duffy blood group sero-phenotype on the PMR, the latter consistent with our observations in vitro using *P*. *knowlesi* parasites transgenic for PvDBP (59). However, Duffy blood group sero-phenotype has been linked to susceptibility of *P*. *vivax* clinical malaria following natural infection (60). Consequently, CHMI studies in larger numbers of volunteers will be required to more stringently assess for any relationships between blood group antigens and the observed PMR and to more accurately establish the natural variability in the PMR observed in malaria-naive adults. A second striking difference between the 2 CHMI models was the apparent minimal gametocytemia following mosquito-bite CHMI, in contrast to blood-stage CHMI. In the latter, the *pvs25* transcripts (a marker of mature female gametocytes) were reliably detected in all 6 volunteers, reaching levels comparable with those reported in other *P*. *vivax* blood-stage CHMI studies (19, 20). Notably, poor transmission to mosquitoes was reported in another *P*. *vivax* mosquito-bite CHMI trial, consistent with our data here (61). Interestingly, a more recent study comparing the same 2 CHMI models with *P*. *falciparum* reported the same finding (17). Why blood-stage CHMI appears to lead to much greater gametocytemia than mosquito-bite CHMI, despite reaching comparable levels of overall parasitemia by the time of diagnosis, remains to be determined. However, this might reflect the greater number of asexual growth cycles since liver egress or a longer time to diagnosis, allowing for an extended window for conversion of asexual parasites.

Finally, we proceeded to undertake a genomic analysis of the new *P*. *vivax* PvW1 clone. The need to drug-treat volunteer infections at relatively low parasitemia limited the amount of PvW1 parasite DNA that could be isolated for sequencing. Nevertheless, a very high–quality genome assembly for PvW1 was created by using a hybrid assembly method, which combined long PacBio reads with short Illumina reads. The PacBio library was created using low-input PacBio technology developed to create a genome assembly from a single mosquito (62), and is — to our knowledge — the first time that this has been applied to *Plasmodium* parasites. Our goal is that the PvW1 clone will become a valuable tool for vaccine discovery, drug testing, and assessment of *P*. *vivax* in vivo immunobiology. Accurate assessment of both the sequence and copy number of vaccine candidate antigens within the PvW1 genome will, thus, be critical in designing future vaccine immunogens and interpreting CHMI efficacy studies. The high quality of the PvW1 assembly allowed us to easily report on leading vaccine candidate antigens, analyze genes and polymorphisms associated with drug resistance in field studies, and resolve 1145 VIR genes, as well as the smaller polymorphic PvMSP3 multigene family. Although the function of the highly variable subtelomeric multigene VIR family is not well defined, related genes are found in high numbers in most *Plasmodium* species that infect humans, monkeys, and rodents, and some are thought to be involved in immune evasion, including by directly binding to and downregulating NK cell ligands (63). Our cluster analysis will now enable comparison of gene function within and between clusters, and it should help in the future elucidation of the function of the VIR gene family.

In conclusion, we have developed a mosquito-transmitted stabilate using a potentially new clonal field isolate of *P*. *vivax* and combined methodologies for parasite isolation and ultra-low input PacBio sequencing to assemble a reference-quality genome for CHMI. This has (a) revealed polymorphisms in leading drug and vaccine targets that can now be functionally tested in vivo with PvW1 and (b) used a hybrid PacBio/Illumina genome assembly technique to identify 1145 unique VIR genes. This will allow for in vivo switching and selection of multigene families to be measured in *P*. *vivax* in the same way as has been done for *P*. *falciparum* (18). This has allowed us to open up many research avenues, and we have used this model to investigate myeloid cell activation, systemic inflammation, and the fate and function of human T cells during a first-in-life *P*. *vivax* infection (64). The PvW1 parasite should prove to be an invaluable resource for the wider malaria community.

## Methods

Supplemental Methods are available online with this article.

### Study design.

VAC068 was a clinical study to assess the safety of controlled human *P*. *vivax* malaria infection through experimental sporozoite inoculation (by mosquito bite) of healthy malaria-naive United Kingdom adults. The study was conducted in the United Kingdom at the Centre for Clinical Vaccinology and Tropical Medicine (CCVTM), University of Oxford (recruitment, follow-up after CHMI, admission for blood donation and treatment), and the Sir Alexander Fleming Building (Infection and Immunity section) Imperial College of Science, Technology and Medicine, London (sporozoite challenge of volunteers, delivered by mosquito bite in the designated category 3 suite). Concurrent primary objectives of the trial were to assess the immune response to primary *P*. *vivax* infection and to assess gametocytemia following infection. Secondary objectives were to obtain up to 250 mL of blood from each infected volunteer and produce a cryopreserved stabilate of iRBC for future use in blood-stage *P*. *vivax* CHMI studies. VAC068 volunteers were admitted to the CCVTM in Oxford according to a clinical/diagnostic algorithm. Following admission, a 250 mL blood sample was collected using aseptic technique, via a whole blood donation kit containing an in-line leukodepletion filter (Leuokotrap WB, Haemonetics Corp.), at room temperature. Antimalarial treatment (60-hour course of artemether/lumefantrine, Riamet; Novartis Pharmaceuticals UK Ltd.) was started immediately after blood donation, followed by a 14-day course of primaquine, 30 mg once daily. Follow-up was out to 5 years to monitor for any signs of relapse. The VAC069A study assessed the safety and infectivity of blood-stage *P*. *vivax* CHMI in healthy malaria-naive United Kingdom adults, through experimental inoculation with the cryopreserved PvW1-infected erythrocytes collected from Donor 1 in VAC068, at 3 different doses. The PvW1 blood-stage inoculum was thawed and prepared under strict aseptic conditions as previously described for *P*. *falciparum* (16), with some modifications. All 6 volunteers were challenged (2 receiving each dose dilution) and followed up at the CCVTM. Diagnostic criteria were based on thick blood film microscopy results and qPCR in the presence or absence of symptoms. Treatment was completed with either a 60-hour course of Riamet or a 48-hour course of Malarone (GlaxoSmithKline), and volunteers followed up for 90 days. Full details of diagnostic criteria and follow-up schedules for both studies, case-finding in Thailand, and preparation of infected mosquitoes for transfer to the United Kingdom are described in Supplemental Methods.

### Participants.

Healthy, malaria-naive males and nonpregnant females aged 18–50 were invited to participate in the study. Two and 6 volunteers were enrolled for each respective trial in total. A full list of inclusion and exclusion criteria, and specific considerations for screening of healthy United Kingdom adult volunteers for the VAC068 study, are reported in Supplemental Methods.

### Safety analysis.

Data on both solicited AEs occurring during and after the CHMI period (that may have related to CHMI or antimalarial treatment), as well as any unsolicited AEs, were collected at clinic visits, from dC+1 up until the end of primaquine antimalarial treatment (VAC068) and until 6 days after initiation of Riamet/Malarone treatment (VAC069A). Volunteers were given a card on which to document the end date of any outstanding malaria symptoms ongoing between completing antimalarial therapy and their next clinic visit. Data on SAEs were collected throughout the entire study period. Details on assignment of severity grading and causality are provided in the Supplemental Methods.

### Total parasite quantification.

qPCR was used to monitor total *P*. *vivax* blood-stage parasitemia in volunteers’ blood in real time. The assay targets the 18S ribosomal RNA (rRNA) gene and was adapted from previously published methodology (19, 53).

### Thick blood film microscopy.

Collection of blood, preparation of thick films, and slide reading for VAC068 volunteers were performed according to Jenner Institute Standard Operating Procedure (SOP) ML009. Briefly, slides were prepared using Field’s stain A and then Field’s stain B. In total, 200 fields at high power (1000×) were read. Visualization of 2 or more parasites in 200 high-power fields constituted a positive result.

### Cryopreservation and in vitro testing of P. vivax–infected blood (VAC068).

After blood donation, the leukodepleted blood from both volunteers was maintained at ~37°C and transported immediately to the Jenner Institute Laboratories. RBC were separated from plasma by centrifugation (830*g* for 5 minutes, set to 37°C) before mixing the RBC with Glycerolyte 57 (Fenwal 4A7833) at 1:2 volume ratio. All procedures were conducted according to SOPs under stringent quality assurance (QA) oversight and guidance from a qualified person (QP) at the University of Oxford. The RBC-Glycerolyte mixture was finally aliquoted at 1.5 mL per cryovial, transferred into CoolCells (Corning, 432009) and placed at –80°C within 2 hours and 30 minutes of blood donation to freeze overnight; the following day, the frozen cryovials were transferred to long-term storage in liquid nitrogen. A final screen for blood-borne infections was conducted on the plasma, derived directly from the blood donation (separated from the RBC prior to cryopreservation), in line with testing procedures performed by the United Kingdom NHS Blood Transfusion service. RNA PCR for HIV-1 and hepatitis C, DNA PCR for hepatitis B and EBV CMV, and serology for HIV-2, HTLV-1, HTLV-2, and Treponema pallidum was performed on thawed plasma samples at University Hospitals Birmingham NHS Foundation Trust, United Kingdom (Public Health England, Birmingham Laboratory). Separately, screening of a blood sample from Donor 1 for the Kell blood group antigen was performed by Oxford University Hospitals NHS Trust Haematology Laboratory. The cryopreserved stabilate from Donor 1 was also tested for sterility by direct inoculation and mycoplasma by specific culture. Finally, endotoxin was quantified by kinetic chromogenic limulus amoebocyte lysate assay. These assays were conducted by a Contract Research Organization: SGS Vitrology (Glasgow, United Kingdom) or SGS Vitrology’s contracted services at Moredun Scientific (Penicuik, Scotland, United Kingdom). The tests were nonregulatory standard and performed for information only.

### Gametocyte quantification.

*P*. *vivax* gametocytemia was determined by 1-step qPCR targeting the messenger RNA marker of female mature gametocytes, pvs25. For RNA extraction, samples were processed within 4 hours of blood sampling (Qiagen), followed by 1-step RT-PCR using Luna Universal Probe One-Step RT-qPCR Kit (New England Biolabs).

### Modeling of PMR.

A qPCR-derived PMR was modeled based on previously described methodology (28, 53, 65).

### Anti-PvDBP_RII standardized ELISA.

ELISAs to quantify circulating PvDBP_RII-specific total IgG responses were performed using standardized methodology, similar to that previously described (29). Day C–1 and dC+90 serum or plasma samples from the VAC068 and VAC069A volunteers were tested, alongside samples from 8 healthy United Kingdom adults previously vaccinated in the VAC051 Phase Ia trial of a candidate PvDBP_RII vaccine (Group 2C) (29).

### Anti-PvMSP1_19_ ELISA.

Anti-PvMSP1_19_–specific total IgG responses were measured in VAC068 volunteer serum or plasma via indirect ELISA (same test samples as for the PvDBP_RII ELISA).

### Illumina and long-read sequencing.

DNA was extracted from blood taken from the VAC068 volunteers at 11 and 14 days after CHMI using the Qiagen blood DNA midi kit and sequenced with Illumina HiSeq X10 with 150 bp paired end reads. See Supplemental Methods for details on preparation of schizonts, high–molecular weight DNA extraction, Shearing and PacBio library construction and sequencing, and VIR gene analysis.

### Data and materials availability.

Requests for materials should be addressed to the corresponding authors. The genome assembly and annotation for PvW1 are available from the European Nucleotide Archive under project accession PRJEB45464.

### Statistics.

Unless otherwise stated, data were analyzed using GraphPad Prism version 9.1.1 for Windows (GraphPad Software Inc.). All tests used were 2 tailed and are described in the text. A value of *P* < 0.05 was considered significant.

### Study approvals.

The VAC068 and VAC069 trials were registered on ClinicalTrials.gov (NCT03377296 and NCT03797989, respectively) and were conducted according to the principles of the current revision of the Declaration of Helsinki 2008 and in full conformity with the ICH guidelines for Good Clinical Practice. All volunteers signed written consent forms, and consent was checked to ensure volunteers were willing to proceed prior to CHMI. The VAC068 study received ethical approval from the United Kingdom NHS Research Ethics Service (Oxfordshire Research Ethics Committee A, Ref 17/SC/0389). The VAC069 study received ethical approval from the United Kingdom NHS Research Ethics Service (South Central — Hampshire A Research Ethics Committee, Ref 18/SC/0577).

## Author contributions

AMM, YT, SES, JRB, AK, DQ, CMN, NJE, TAR, FRL, WR, KJDE, JSC, TDO, AJR, FAB, GMCL, IDP, AM, MZ, MM, RLR, MB, CHM, JCS, NR, CK, NM, CS, TP, KM, DJR, AMB, PJS, JS, SB, JCR, and SJD conceived and performed the experiments. AMM, YT, SES, JRB, AK, NJE, KJDE, JSC, EA, TDO, AJR, JCS, MMH, BK, JS, SB, JCR, and SJD analyzed the data. AML, FLN, KJJ, and IJT performed project management. AMM, AK, SES, JRB, JCR, and SJD wrote the paper.

## Supplementary Material

Supplemental data

## Figures and Tables

**Table 1:**
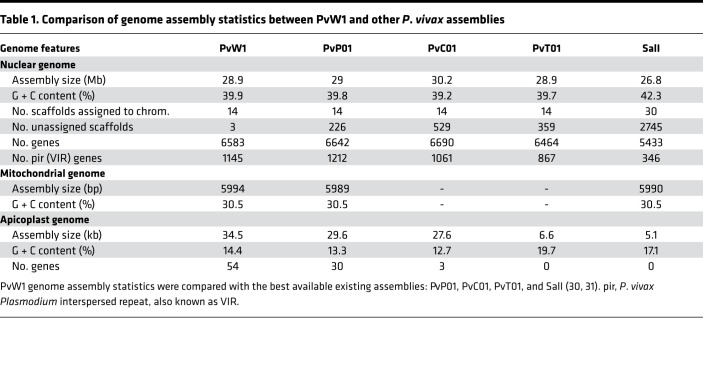
Comparison of genome assembly statistics between PvW1 and other *P*. *vivax* assemblies

## Version 1 10/05/2021

In-Press Preview

## Version 2 12/08/2021

Electronic publication
