# Supplementary material for: Controlled human malaria infection with a clone of Plasmodium vivax with high-quality genome assembly
Source: JCI Insight. 2021 Dec 8;6(23):e152465. doi: 10.1172/jci.insight.152465 (PMC8675201; doi:10.1172/jci.insight.152465)
Supplement: Supplemental data [file jciinsight-6-152465-s118.pdf]

Supplementary Methods

Thailand: Source patient case-finding and preparation of infected mosquitoes

For infection of mosquitoes, source patients were recruited from a medical clinic in Songkhala, one of Thailand's southern *Plasmodium vivax* endemic areas; this protocol was approved by the Ethical Committee of The Faculty of Tropical Medicine, Mahidol University, Thailand (protocol number TMEC 18-014). Patients were consented to having a 20 mL blood sample taken for blood-borne infection testing, endemic mosquito-borne infection testing and *P. vivax* diagnostic testing. *P. vivax* was first diagnosed by microscopy at the field site, then confirmed by microscopy and nested PCR analysis following transport of the blood samples to the Mahidol Vivax Research Unit (MVRU), Mahidol University, Bangkok, to rule out the presence of any other *Plasmodium* species (data not shown). As *Anopheles* species are also known vectors of *Wucherichia bancrofti,* the main causative agent of lymphatic filariasis, source patient blood was also screened for filarial disease via rapid diagnostic test for IgG4 antibodies to the *W. bancrofti* Wb123 antigen (Standard Diagnostics, Inc.); all tests were negative.

Approximately 5 mL of each 20 mL blood sample was used to feed up to 3000 laboratory-bred *Anopheles dirus* mosquitoes via a direct membrane feeding system at MVRU. These mosquitoes were previously reared in the laboratory and fed only on rigorously screened human blood (purchased from the Red Cross) to maintain the colony and induce egg-laying. Mosquito infectivity was confirmed at 6-7 days post-feeding via oocyst counts following dissection of the midgut from representative mosquitoes.

In parallel to the mosquito-feeding, and in real-time, the remaining ~15 mL of the source patients' serum and whole blood samples were shipped from Thailand to the UK. These underwent additional testing for blood-borne infections and mosquito-borne diseases other than malaria. For maximal assurance of safety, serological tests for human immunodeficiency virus-1 (HIV-1) and HIV-2, human T cell lymphotropic virus-1 (HTLV-1) and HTLV-2, hepatitis B and C and syphilis were performed at Oxford University Hospitals NHS Trust, Oxford, UK. Alongside these, whole blood from the source patients were also screened for Japanese B encephalitis virus and chikungunya virus by PCR at the Rare Imported Pathogens Laboratory (RIPL) in the UK, due to anecdotal reports of both arboviral infections in *Anopheles* species (1) and in line with the protocols followed by WRAIR in their previous *P. vivax* CHMI study (2). Finally, as a further precautionary measure, although *Anopheles* species are not known to be vectors of dengue, Zika or West Nile viruses, PCR for these

infections were also performed at RIPL on the source patients' blood samples. All source patient infection screen tests were negative.

Alongside infection testing, molecular speciation of *P. vivax* was re-confirmed for each sample by nested PCR using whole blood and an in-house research-grade laboratory assay method, adapted from (3), at the University of Oxford. Positive control DNA samples for different *Plasmodium* parasite species were a kind gift from Prof Colin Sutherland (LSHTM, UK). Each sample was also genotyped to measure multiplicity of *P. vivax* infection; here extracted DNA was processed by the Wellcome Sanger Institute in Cambridge, UK using a SNP barcode panel as described by the MalariaGEN network (4), with detailed methods available at https://www.malariagen.net/resource/29.

The batch of mosquitoes fed off blood from patient C05-001 was ultimately selected for use in the VAC068 CHMI trial, and was shipped from MVRU in Thailand to Imperial College London, UK. Shipment took less than 48 hours, and no mosquito mortality was observed.

VAC068: Objectives

VAC068 was a clinical study to assess the safety of controlled human *P. vivax* malaria infection through experimental sporozoite inoculation (by mosquito-bite) of healthy malaria-naïve UK adults, and to characterize parasite growth and immune responses. The study was conducted in the UK at the Centre for Clinical Vaccinology and Tropical Medicine (CCVTM), University of Oxford (follow-up post-CHMI, admission for blood donation and treatment) and at the Sir Alexander Fleming Building (Infection and Immunity section) Imperial College of Science, Technology and Medicine, London (sporozoite challenge of volunteers). Recruited volunteers were healthy, malaria-naïve adults (male and female) aged between 18 and 50 years.

The primary objectives of the trial were i) to assess the safety and feasibility of *P. vivax* sporozoite CHMI (via mosquito-bite) in two healthy human volunteers; ii) to assess the immune response to primary *P. vivax* infection delivered by mosquito bite; and iii) to asses gametocytemia following primary *P. vivax* infection delivered by mosquito bite. Secondary objectives were to obtain up to 250 mL of blood from each infected volunteer and produce a cryopreserved stabilate of parasite-infected human red blood cells (iRBC) for future use in blood-stage *P. vivax* CHMI studies.

VAC068: Specific considerations for screening of healthy UK adult volunteers

Two healthy UK adult volunteers were consented and enrolled into the VAC068 trial. Alongside the routine screening and inclusion/exclusion criteria used for CHMI trials at the University of Oxford

site (see below), these volunteers were also specifically screened to be: i) blood group O rhesus-negative (O-), i.e. universal donors suitable for production of the cryopreserved iRBC stabilate; ii) Duffy-blood group positive, to ensure successful *P. vivax* blood-stage infection (5, 6); iii) glucose-6-phosphate dehydrogenase (G6PD) normal, to ensure no hemolytic anemia following curative treatment with primaquine (7); iv) cytochrome P450 2D6 (CYP2D6) genotype predicted to be an extensive metabolizer phenotype (8), to minimize chance of primaquine drug treatment failure against hypnozoites (9), and v) able to satisfactorily metabolize primaquine after administration of a 30 mg test dose (9).

Blood group (ABO, Rhesus and Duffy) were characterized and G6PD activity levels were measured in the NHS Hematology Laboratory at Oxford University Hospitals NHS Trust, UK. CYP2D6 genotype testing was performed by PharmGenomics GmbH, Germany, using the GenoChip method and NCBI reference sequence NG_008376.3. Classification was done according to (8). Measurement of the pharmacokinetic parameters of primaquine was performed by the Division of Experimental Therapeutics, Drug Metabolism and Disposition, at the Walter Reed Army Institute of Research, USA. Plasma samples from both volunteers, taken from 0 to 24 h after a single dose of primaquine (PQ), were analyzed by Liquid Chromatography-Mass Spectrometry (LC-MS) for levels of PQ and its major metabolite, carboxyprimaquine (cPQ). All plasma samples were frozen at -80 °C in laboratory facilities until ready for analysis. Calibration and quality control samples were prepared by spiking blank human plasma with the analyte of interest. Calibration, quality control, and study samples were extracted using a 2x volume of acetonitrile containing an internal standard (mefloquine, MQ). The ratio of the peak area of the analyte to the peak area of the internal standard was used for calibration and interpolation of sample concentrations. LC-MS methodology has been previously described in detail (9, 10).

Each volunteer also underwent an extensive screen for blood-borne infections, performed in line with the Joint UK Blood Transfusion and Tissue Transplantation Services Professional Advisory Committee guidelines (11), in the microbiology laboratory at OUH NHS Trust. Testing comprised serological tests for HIV-1 and HIV-2, hepatitis B and C, syphilis (anti-treponemal antibody), and HTLV-1 and HTLV-2 at screening; and nucleic acid amplification tests for HIV-1 and hepatitis B and C, as well as repeat serological tests for HTLV-1 and HTLV-2 and syphilis 7 days before challenge. In addition, the volunteers were screened serologically for Epstein-Barr virus (EBV) and cytomegalovirus (CMV). However, given i) both of these viruses are cell-associated, being carried within leukocytes, and the risk of transfusion-induced CMV and EBV infection has been deemed to be minimal following leukodepletion (12); and ii) the historical experience of us and others with a *P.*

falciparum 3D7 clone blood-stage inoculum that originated from a CMV and EBV seropositive donor (13, 14), we did not exclude volunteers based on their serostatus for these two viruses.

VAC068: Other routine screening, and full list of inclusion and exclusion criteria

For the two VAC068 volunteers, a medical history and physical examination were conducted at the screening visit. Hematology screening bloods included a full blood count, and a hemoglobinopathy screen was performed retrospectively post-challenge; whilst biochemistry measurements at screening included urea and electrolytes, liver function tests, magnesium & cholesterol. Dipstick urinalysis for all volunteers and pregnancy testing for female volunteers were conducted at screening, as well as an electrocardiogram. Pregnancy testing (in the form of serum beta human chorionic gonadotrophin, BHCG) was also carried out in female volunteers the day before CHMI (dC-1), and then at 7 days post-CHMI (dC+7), dC+14, just prior to starting primaquine treatment and at dC+21. The full list of inclusion and exclusion criteria is shown below:

Inclusion criteria: Volunteers had to satisfy all the following criteria to be eligible for the study:

- Healthy adult aged 18 to 50 years.
- Blood group O, Rhesus negative.
- Red blood cells positive for the Duffy antigen/chemokine receptor (DARC).
- High metabolizer of primaquine (as determined by CYP2D6 genotype).
- Normal serum levels of glucose-6-phosphate dehydrogenase (G6PD).
- Satisfactory serum levels of primaquine (when administered as test dose).
- Able and willing (in the Investigator’s opinion) to comply with all study requirements.
- Willing to allow the Investigators to discuss the volunteer’s medical history with their General Practitioner.
- Women only: Must practice continuous effective contraception for the duration of the clinic visits (first 3 months post-CHMI).
- Agreement to refrain from blood donation during the course of the study and for at least 5 years after the end of their involvement in the study.

- Written informed consent to participate in the trial.
- Reachable (24/7) by mobile phone during the period between CHMI and completion of all antimalarial treatment.
- Willing to take a curative anti-malaria regimen following CHMI.
- Willing to be admitted to the research bay at the CCVTM on the Churchill Hospital, Oxford site for blood donation and clinical monitoring, until antimalarial treatment is underway and their symptoms are settling.
- Willing to reside in Oxford for the duration of the study, until all antimalarials have been completed.
- Answer all questions on the informed consent quiz correctly.

Exclusion Criteria

Volunteers were not eligible to participate if any of the following applied:

- History of clinical malaria (any species).
- Travel to a clearly malaria endemic locality during the study period or within the preceding six months.
- Use of systemic antibiotics with known antimalarial activity within 30 days of CHMI (e.g. trimethoprim-sulfamethoxazole, doxycycline, tetracycline, clindamycin, erythromycin, fluoroquinolones and azithromycin).
- Blood group A/B and/or Rhesus positive.
- Red blood cells negative for the Duffy antigen/chemokine receptor (DARC).
- Glucose-6-phosphate dehydrogenase (G6PD) deficient.
- Inadequate serum levels of primaquine (when administered as test dose).
- Current anemia (hemoglobin < 9 g/dL).

- Use of immunoglobulins or blood products (e.g., blood transfusion) at any time in the past.
- History of sickle cell anemia, sickle cell trait, thalassemia or thalassemia trait or any hematological condition that could affect susceptibility to malaria infection.
- Venepuncture unlikely to allow a 250 mL blood donation (as determined by the Investigator).
- Receipt of an investigational product in the 30 days preceding enrolment, or planned receipt during the study period.
- Prior receipt of an investigational vaccine likely to impact on interpretation of the trial data or the *P. vivax* parasite as assessed by the Investigator.
- Any confirmed or suspected immunosuppressive or immunodeficient state, including HIV infection; asplenia; recurrent, severe infections and chronic (more than 14 days) immunosuppressant medication within the past 6 months (inhaled and topical steroids are allowed).
- History of allergic disease or reactions likely to be exacerbated by malaria infection.
- Pregnancy, lactation or intention to become pregnant during the study.
- Use of medications known to cause prolongation of the QT interval ***and*** existing contraindication to the use of Malarone.
- Use of medications known to have a potentially clinically significant interaction with Riamet® ***and*** Malarone.
- Any clinical condition known to prolong the QT interval.
- History of cardiac arrhythmia, including clinically relevant bradycardia.
- Disturbances of electrolyte balance, e.g. hypokalemia or hypomagnesemia.
- Family history of congenital QT prolongation or sudden death.
- Contraindications to the use of both of the proposed anti-malarial medications; Riamet® Malarone.

- Contraindications to the use of primaquine.
- History of cancer (except basal cell carcinoma of the skin and cervical carcinoma in situ).
- History of serious psychiatric condition that may affect participation in the study.
- Any other serious chronic illness requiring hospital specialist supervision.
- Suspected or known current alcohol abuse as defined by an alcohol intake of greater than 42 standard UK units every week.
- Suspected or known injecting drug abuse in the 5 years preceding enrolment.
- Hepatitis B surface antigen (HBsAg) detected in serum.
- Seropositive for HTLV-1 or -2 (antibodies to HTLV) at screening or at dC-7.
- Seropositive for hepatitis C virus (antibodies to HCV) at screening or at dC-7 (***unless*** has taken part in a prior hepatitis C vaccine study with confirmed negative HCV antibodies prior to participation in that study, and negative HCV RNA PCR at screening for this study).
- Seropositive for RPR (antibodies to syphilis) at screening or at dC-7.
- Detectable HIV or hepatitis C virus by PCR at dC-7.
- Positive family history in both 1st AND 2nd degree relatives < 50 years old for cardiac disease.
- Volunteers unable to be closely followed for social, geographic or psychological reasons.
- Any clinically significant abnormal finding on biochemistry or hematology blood tests, urinalysis or clinical examination. In the event of abnormal test results, confirmatory repeat tests will be requested.
- Any other significant disease, disorder, or finding which may significantly increase the risk to the volunteer because of participation in the study, affect the ability of the volunteer to participate in the study or impair interpretation of the study data.

VAC068: Mosquito-bite CHMI at Imperial College, London

Sporozoite CHMI delivered by mosquito-bite was conducted in the designated category 3 (CL3) suite within the Sir Alexander Fleming Building (SAF) at Imperial College London, UK. Mosquitoes were supplied directly from Thailand via World Courier in a temperature-controlled box at 28 ^{o}C with receipt acknowledged by an appropriate researcher. Infected mosquitoes were immediately transferred to a secure room within the insectary and maintained on 10 % fructose until 24 hours prior to skin feeding. Prior to CHMI, an appropriately trained researcher prepared the mosquitoes into secure small pots that were brought to the volunteers as described in a Standard Operating Procedure (SOP). The two healthy UK adult volunteers screened and consented to take part in VAC068 were each exposed to five "infectious bites" under controlled conditions. Here, an infectious mosquito bite was defined post-skin feeding by microscopic examination, with confirmation of >10 sporozoites in the mosquito's salivary glands as well as the presence of human blood in the midgut.

VAC068: Participant follow-up post-CHMI

The two VAC068 volunteers were reviewed by telephone daily for the first 5 days post-CHMI (dC+1 to dC+5), then reviewed in clinic on the evening of day 6 post-CHMI (dC+6.5) and subsequently twice daily (morning/evening) to monitor for symptoms/signs of malaria and check for development of parasitemia by quantitative PCR (qPCR) and thick blood film microscopy.

Volunteers were admitted to the clinical trial unit at the CCVTM in Oxford according to a clinical / diagnostic algorithm. This stipulated admission at an absolute threshold parasitemia of >10,000 gc/mL OR at threshold of >2,000 gc/mL in the presence of significant malaria symptoms. Following admission, the protocol allowed for a 72 hour window in which to donate blood; volunteer 01-008 donated blood first at dC+14 and volunteer 01-004 at dC+14.5. A 250 mL blood sample was collected using aseptic technique, via a whole blood donation kit containing an in-line leukodepletion filter (Leuokotrap WB, Haemonetics Corp), at room temperature. The blood donation kit was placed on an agitator (Blood Collection Monitor and Mixer, HemoFlow 400^{TM}, Applied Science UK Ltd). This maintained automatic mixing of the blood during flow at a rate of >30mL/min, ensuring combination with the contained anticoagulant and so minimizing the risk of coagulation. In order to anonymize the blood donor, these samples were randomized and relabelled either "Donor 1" or "Donor 2". Traceability of the blood donor is, however, maintained within a confidential clinical record which may be accessed by the trial Chief Investigator on behalf of the trial Sponsor if deemed necessary for safety reasons.

Antimalarial treatment (60-hour course of artemether/lumefantrine, Riamet®) was started immediately after blood donation, followed by a 14-day course of primaquine, 30 mg once daily. Both volunteers attended clinic on alternate mornings for directly observed primaquine treatment (telephoned to confirm consumption on intervening days) until they had completed the course.

Final follow-ups in clinic were performed at dC+45 and at dC+90, and between these days volunteers were contacted fortnightly by email (on dC+59 and dC+73) to ensure they remained well and asymptomatic. Both volunteers also underwent repeat serological testing for HIV-1, HIV-2, hepatitis B and C, syphilis, HTLV-1 and HTLV-2 at dC+90 to ensure that no seroconversion from a recently-acquired infection (that may have been undetectable around the time of CHMI) had occurred since the challenge period. Subsequently the volunteers received an email from the Investigators fortnightly up until one year post-CHMI and then annually from 1-5 years post-CHMI (ongoing). This was to enquire about the presence of any symptoms suggestive of *P. vivax* malaria relapse (or any medical intervention sought/received) since they were last seen.

Total parasite quantification

Quantitative PCR (qPCR) was used to monitor total *P. vivax* blood-stage parasitemia in volunteers' blood in real-time. The assay targets the 18S ribosomal RNA (rRNA) gene and was adapted from previously published methodology (15, 16). DNA was initially extracted from 0.4 mL whole EDTA blood using the Qiagen DSP DNA Blood mini Kit. 5 % of each extraction (total eluate volume = 100 µL, with 5 µL used per assay) was run in triplicate for qPCR; equivalent to 60 µL blood directly assessed. An additional extraction was performed post-CHMI on aliquots of frozen blood from all time-points, using a QIAsymphony SP robot, utilizing the Qiagen DSP Blood Midi Kit and the pre-loaded Blood 400 v6 extraction protocol, with a 100 µL elution in ATE buffer selected, (giving identical extraction and elution volumes in both manual and automated extractions). Both methods had been shown to be equivalent, but with greater ease of use and reduced chance of cross contamination with the automated extraction. Additionally, aliquots of dC-1 samples were spiked with a known concentration of positive control DNA to check there was no presence of PCR inhibitors in volunteers' blood prior to CHMI.

Following DNA extraction, a standard Taqman absolute quantitation was used against a standard curve to amplify a 183 bp PCR product from the multi-copy, highly conserved 18S ribosomal RNA genes of *Plasmodium* spp. qPCR using the following adapted oligonucleotide primers and probe (15): 18s forward primer 5'-AGG AAG TTT AAG GCA ACA ACA GGT-3', 18s reverse primer 5'-GCA ATA ATC TAT CCC CAT CAC GA-3' and shortened FAM labelled probe sequence 5'-TGA ACT AGG CTG CAC GCG-3', was run on an ABI StepOne Plus machine with v2.3 software. Default

Universal qPCR (target FAM-NFQ-MGB) and QC settings were used apart from the use of 40 cycles and 25 μL reaction volume.

This qPCR detects DNA from pan-*Plasmodium* species, but unlike the synchronous growth of *P. falciparum*, circulating *P. vivax* iRBC may contain up to 10-15 individual genomes (in blood-stage late trophozoites and schizonts) and can also include the presence of gametocytes. The qPCR score is therefore reported in genome copies/mL (gc/mL) as opposed to a quantity of parasites.

The standard curve was generated from dilution of a linearized plasmid encoding part of the *Plasmodium* spp. 18S ribosomal RNA gene and calibrated using known *P. falciparum* spiked blood samples initially and then reference DNA extracted from whole blood from *P. vivax*-infected patient samples in Thailand where parasites had been quantified by microscopy (kindly provided by Mahidol University, Bangkok, Thailand). Based upon earlier results obtained using dilution series of microscopically-counted cultured *P. falciparum* (Pf) parasites, a Pf-specific 18S rRNA Taqman qPCR showed a lower limit of quantification (LLQ, defined as %CV <20%) of around 20 Pf parasites (p)/mL blood (17). Counted parasite dilution series results also suggested that the lower limit of probable detection (LLD, i.e. a probability of >50% of ≥1 positive result among three replicate qPCR reactions) is in the region of 5 p/mL, while samples at 1 p/mL are consistently negative (24/24 qPCR reactions). Positive results in this assay (even at very low level) are thus essentially 100 % specific for genuine parasitemia, with positive results beneath the LLQ likely to signify parasitemia in the range 2-20 p/mL. Similar sensitivity in terms of genome copy detection was observed when using the pan-*Plasmodium* qPCR described above and the diluted *P. vivax*-infected patient blood test samples from Thailand. As noted, these samples had microscopically mixed life stages with varying copies of the 18S rRNA gene and thus the assay readout is reported in terms of gc/mL. Based on this and the above experiments, 20 gc/mL was set as the minimum level to meet positive reporting criteria, but all raw data are shown in the Results.

For quality control purposes, qPCR samples were re-tested if;

- Replicates included a mixture of positive and negative (in terms of amplification) results with one or more positive results > 100 gc/mL;
- The % CV of any results were high outliers.

All ‘passed’ data following the quality control steps above, including any 0 values, were used to generate the final mean qPCR result for each time-point.

Thick blood film microscopy

Collection of blood, preparation of thick films and slide reading were performed according to Jenner Institute Standard Operating Procedure (SOP) ML009. Briefly, slides were prepared using Field's stain A and then Field's stain B. 200 fields at high power (1000x) were read. Visualization of 2 or more parasites in 200 high power fields constituted a positive result. For internal quality control, all slides were read separately by two experienced Thai microscopists, with a third read if results were discordant.

VAC068: Cryopreservation and *in vitro* testing of *P. vivax* infected blood

After blood donation, the leukodepleted blood from both volunteers was maintained at ~37 ^{o}C and transported immediately to the Jenner Institute Laboratories, University of Oxford. Here, RBC were separated from plasma by centrifugation before mixing the RBC with Glycerolyte 57 (Fenwal 4A7833) at 1:2 volume ratio. All procedures were conducted according to SOPs under stringent Quality Assurance (QA) oversight and guidance from a Qualified Person (QP) at the University of Oxford. The RBC-Glycerolyte mixture was finally aliquoted at 1.5 mL per cryovial, transferred into CoolCells (Corning 432009) and placed at -80 ^{o}C within 2 h 30 min of blood donation to freeze overnight; the following day the frozen cryovials were transferred to long-term storage in liquid nitrogen.

A final screen for blood-borne infections was conducted on the plasma, derived directly from the blood donation (separated from the RBC prior to cryopreservation), in line with testing procedures performed by the UK NHS Blood Transfusion service. RNA PCR for HIV-1 and hepatitis C, DNA PCR for hepatitis B, EBV CMV, and serology for HIV-2, HTLV-1, HTLV-2, and *Treponema pallidum* was performed on thawed plasma samples at University Hospitals Birmingham NHS Foundation Trust, UK (Public Health England, Birmingham Laboratory). Separately, screening of a blood sample from Donor 1 for the Kell blood group antigen was performed by Oxford University Hospitals NHS Trust Haematology Laboratory, UK.

The cryopreserved stabilate from Donor 1 was also tested for sterility by direct inoculation and mycoplasma by specific culture; both tests were negative. In addition, endotoxin was quantified by kinetic chromogenic limulus amoebocyte lysate assay, reporting a result <2 EU/mL. These assays were conducted by a Contract Research Organization: SGS Vitrology, Glasgow, UK or SGS Vitrology's contracted services at Moredun Scientific, Penicuik, Scotland, UK. The tests were non-regulatory standard and performed for information only.

Parasite viability assay

A vial of the cryopreserved stabilate was gently warmed at 37 °C, before addition of 0.2x volume 12 % NaCl dropwise. After 5 min incubation, the cells were pelleted at 1500 x*g* for 5 min. Supernatant was removed, before addition of 10x volume 1.6 % NaCl dropwise followed by 10x volume 0.9 % NaCl. The cells were then immediately spun down as before and the pelleted RBC transferred to short term *in vitro* culture. Here, the sample was added to McCoy 5A medium (Sigma) supplemented with 2.4 g/L D-glucose, 25 mM HEPES and 200 mM hypoxanthine (all from Sigma, St. Louis) and 20 % heat-inactivated human O serum, in an atmosphere of 5 % O_2 at 37.5 ˚C. No exogenous cells were added to the parasite viability assay as parasitemia in both donors was low. At each time point, a 20 µL aliquot of RBC was taken for DNA extraction and parasite genome copies quantified by qPCR. In addition, 10 µL RBC were used to make thick and thin blood smears for parasite growth and morphology monitoring by light microscopy. Here, Giemsa stain (Sigma, St. Louis) was filtered using a 0.8 µm filter (Merck Millipore, Ireland) then diluted to 5 % using water, prior to staining the slides at RT for 20 min before gently washing and drying the slides

VAC069: Objectives

The subsequent VAC069 trial assessed the safety and feasibility of blood-stage *P. vivax* CHMI through experimental inoculation with the cryopreserved PvW1 infected erythrocytes (collected in VAC068) in healthy malaria-naïve UK adults. The VAC069 trial is an on-going multi-part study, and the work reported here covers the first part of this trial in six volunteers (termed "VAC069A"). All six volunteers were challenged and followed up at the CCVTM, Oxford, UK.

The VAC069A trial tested safety and infectivity of the PvW1 cryopreserved stabilate (from Donor 1 in VAC068) by blood-stage CHMI, in line with prior experience using the *P. falciparum* blood-stage CHMI model (13, 14, 18). This proof-of-concept clinical trial sought to assess feasibility of infection at three different doses of PvW1 blood-stage inoculum. Two volunteers receive a whole vial's worth of iRBC ("neat"), two volunteers received one fifth of the challenge dose via a 1:5 dilution, and the final two volunteers were inoculated with one twentieth of the dose via a 1:20 dilution.

VAC069A: Study population and screening

This study recruited healthy, malaria-naïve adult volunteers (male and female) aged between 18 and 50 years. The inclusion and exclusion criteria for the VAC069A study were very similar to those of VAC068, with the only differences being removal of criteria related to ABO/Rhesus blood group, G6PD activity, CYP2D6 genotype and primaquine metabolism (as not relevant to blood-stage CHMI)

and removal of viral serology at dC-7 (as these volunteers were not donating blood for use in future clinical studies). Duffy blood group positivity (and serological phenotype) was also confirmed. Screening hemoglobin cut-offs were also more conservative (Hemoglobin <120 g/L for a female volunteer or <130 g/L for a male volunteer prior to primary CHMI) to minimize the chances of anemia resulting from cumulative blood volume taken over the whole VAC069 trial period.

VAC069A: Blood-stage inoculum preparation and CHMI

The PvW1 blood-stage inoculum was thawed and prepared under strict aseptic conditions as previously described for *P. falciparum* (14), with some modifications. Briefly, five vials of cryopreserved erythrocytes (containing approximately 0.5mL of red blood cells each) were thawed in parallel in a derogated containment level III laboratory area using solutions licensed for clinical use and single-use disposable consumables. A class II microbiological safety cabinet (MSC) was used to prepare the inoculum, which was fumigated with hydrogen peroxide and decontamination validated prior to use. To prepare the inoculum, 0.2 volume 12 % saline was added dropwise to the contents of each (~1.5 mL) vial of thawed infected blood. Each sample was left for 5 min, before an additional 10 volumes of 1.6 % saline was added dropwise prior to centrifugation for 4 min at 800 x*g*. Each supernatant was removed, and 10 mL of 0.9% saline was added dropwise. The cell pellets were then washed twice in 0.9 % saline before a final resuspension in 0.9 % saline. At this final step, the five samples (from the five original cryovials) were combined into one 10 mL sample in 0.9 % saline. This 10 mL suspension was then divided into aliquots, equivalent to one original cryovial (i.e. 2mL), one fifth of a cryovial, and one twentieth of a cryovial by further dilution in 0.9 % saline. Each dosing aliquot was made up to a total volume of 5 mL in 0.9 % saline in a sterile syringe for injection and transported to the clinic. Retrospective qPCR analysis indicated the neat inoculum dose to contain 2322 gc in total (although this is likely to contain a mixture of live and dead parasites post-thawing).

The reconstituted blood-stage inoculum (5 mL per syringe) was injected intravenously via an indwelling cannula, preceded and followed by a saline flush. Single volunteers from each of the three dosing groups were administered the inoculum first. The inoculum was subsequently administered to the remaining three volunteers (one from each dosing group). All six volunteers were inoculated within 2 h 24 min of thawing the cryopreserved stabilate. Volunteers were then observed for 1 h before discharge from the clinical facility. Following CHMI, a leftover sample of the inoculum was also cultured and shown to be negative for bacterial contamination.

VAC069A: Participant follow-up post-CHMI

The VAC069A volunteers were reviewed in clinic once in the morning of day 1 post-CHMI (dC+1), then twice daily from day 2 until day 12 post-CHMI inclusive (dC+2 to dC+12.5). From dC+13 to dC+20.5, visits were either once or twice daily depending on the qPCR result. Once a qPCR threshold of 1000 gc/mL was reached, visits continued twice daily. If qPCR had not reached this threshold, visits reduced to once daily. Diagnostic criteria were based on thick blood film microscopy results and qPCR in the presence or absence of symptoms:

- If symptomatic: ≥2 parasites visible on 200 fields (thick blood film microscopy) OR a parasitemia of >5,000 gc/mL by qPCR.
- If asymptomatic: a parasitemia of >10,000 gc/mL on qPCR OR a parasitemia of >5,000 gc/mL PLUS ≥2 parasites visible on 200 fields (thick blood film microscopy).

Treatment was completed with either a 60-hour course of Riamet® or a 48-hour course of Malarone. Volunteers were directly observed taking their 24- and 48-hour doses (T+1 and T+2 visits, respectively). Subsequent follow-ups visits in clinic were 6 days post-initiation of antimalarial treatment (T+6), 28 days, 45 days and 90 days post-CHMI.

VAC068 and VAC069A safety analysis

Data on both solicited AEs occurring during and after the CHMI period (that may have related to CHMI or antimalarial treatment) as well as any unsolicited AEs, were collected at clinic visits, from dC+1 up until the end of primaquine antimalarial treatment (VAC068) and until 6 days post-initiation of Riamet®/Malarone treatment (VAC069A). Volunteers were given a card on which to document the end date of any outstanding malaria symptoms on-going between completing anti-malarial therapy and their next clinic visit (dC+45 in VAC068, and dC+28 in VAC069A).

Data on serious adverse events (SAEs) were collected throughout the entire study period (5 years for VAC068, 3 months for VAC069A).

Volunteers graded all AEs as mild, moderate or severe:

- **GRADE 0:** None.
- **GRADE 1:** Transient or mild discomfort (< 48 h); no medical intervention/therapy required.
- **GRADE 2:** Mild to moderate limitation in activity – some assistance may be needed; no or minimal medical intervention/therapy required.

- **GRADE 3:** Marked limitation in activity, some assistance usually required; medical intervention/therapy required; hospitalization possible.

For each unsolicited AE, an assessment of the relationship of the AE to the study intervention (CHMI/ antimalarial treatment) was undertaken. Alternative causes of the AE, such as the natural history of pre-existing medical conditions, concomitant therapy, other risk factors and the temporal relationship of the event to the study intervention were considered. The likely causality of all unsolicited AEs was assessed as per the criteria below:

- **No Relationship:** No temporal relationship to study intervention ***and*** alternate aetiology (clinical state, environmental or other interventions); ***and*** does not follow known pattern of response to study intervention.
- **Unlikely:** Unlikely temporal relationship to study intervention ***and*** alternate aetiology likely (clinical state, environmental or other interventions) ***and*** does not follow known typical or plausible pattern of response to study intervention.
- **Possible:** Reasonable temporal relationship to study intervention; ***or*** event not readily produced by clinical state, environmental or other interventions; ***or*** similar pattern of response to that seen with other similar interventions.
- **Probable:** Reasonable temporal relationship to study intervention; ***and*** event not readily produced by clinical state, environment, or other interventions ***or*** known pattern of response seen with other similar interventions.
- **Definite:** Reasonable temporal relationship to study intervention; ***and*** event not readily produced by clinical state, environment, or other interventions; ***and*** known pattern of response seen with other similar interventions.

AE data also included the results of hematology (full blood count) and biochemistry (liver function tests, urea and electrolytes) carried out at dC+9, dC+11, within 12 hours of starting antimalarials after blood donation (C+14-14.5), and then at dC+45 and dC+90 (VAC068) and at dC+14, day of diagnosis, T+1, T+6, dC+28 and dC+90 (VAC069A).

Gametocyte quantification

P. vivax gametocytemia was determined by one-step quantitative reverse transcription PCR (qRT-PCR) targeting the messenger RNA marker of female mature gametocytes, *pvs25*. For RNA extraction samples were processed within 4 h of blood sampling: here 50 µL whole blood was mixed with 250 µL RNA protect reagent (Qiagen) for RNA stabilization, until the blood was lysed and had turned black. Samples were then stored at -20 °C. Subsequently, 600 µL D-PBS with 1 % β-ME was

added to each sample and then centrifuged for 15 min at 15,000 x*g*, the supernatant removed and the pellet resuspended in 300 µL RLT lysis buffer (Qiagen) + 1 % β-ME with 20 µL proteinase K and incubated at 55ºC for 10 min. The samples were homogenized into a QIAshredder column (Qiagen) and purified through an RNeasy mini spin column (Qiagen) as per the manufacturer's instructions. Each sample was then eluted in 100 µL RNAse-free water. Thereafter, one-step RT-PCR was performed using Luna® Universal Probe One-Step RT-qPCR Kit (New England Biolabs). Briefly, 5 µL RNA extract was added to a final reaction volume of 25 µL consisting of 12.5 µL Luna® Universal qPCR Master Mix, 0.625 µL Pv25 MGB-FAM probe (5'-CCA ATC CAG AAG ATG AGA-3'), 1.25 µL of each primer (diluted at 10 µM), and 3.375 µL nuclease-free water and 1 µL reverse transcriptase (RT) enzyme (NEB). The *pvs25* primer sequences were 5'-GTT GCT CAT GTG CTA TTG-3' for the forward primer and 5'-CAG ACT TCA TTA TCT GTG TTA-3' for the reverse primer. Analyses were performed on a StepOne Plus machine (Thermo Fisher Scientific) using the StepOne software v2.3. The thermal conditions consisted of reverse transcription (55 °C for 10 min), enzyme activation (95 °C for 1 min) and two-temperature cycling steps (95 °C for 10 s, 60 °C for 1 min, for 45 cycles). All samples were tested in triplicate. Controls without RT enzyme were added to exclude false positives due to the presence of genomic DNA and were tested in duplicate. Ct values were converted into *pvs25* transcript/µL using plate-specific standard curves, generated by serial-diluted *pvs25* RNA transcripts (10^7 - 10 copies/µL). Final values were multiplied by 2 (dilution factor at the RNA extraction step) to report per µL of the original blood sample. *pvs25* RNA transcripts were produced by amplification of the *pvs25* gene by T7 polymerase (HiScribe T7 High Yield RNA Synthesis Kit); RNA purified by lithium chloride extraction before treatment with DNAse I (Qiagen) to eliminate residual DNA; standards were diluted in DNA-free H_2O containing a background of 5 % aspecific human RNA (isolated from blood of a healthy donor), to improve linearity of the standard curve.

Modelling of PMR – VAC068

A qPCR-derived parasite multiplication rate (PMR) was modelled based on previously described methodology (16, 17, 19). In brief, to model the PMR, the arithmetic mean of the three replicate qPCR results obtained for each individual at each time-point was used for model-fitting. Negative individual replicates were assigned a value of 0 gc/mL for the purposes of calculating the arithmetic mean of triplicates (where at least one of the three readings was positive). All qPCR data points which, based upon the mean of the three replicates, were >5 gc/mL were used for modelling and any values ranging from 1-5 gc/mL were replaced with 5 gc/mL. The time interval between the morning and evening bleeds used for qPCR monitoring was set as 0.3 days. PMR per 48 hours was then calculated using a linear model fitted to $\log_{10}$-transformed qPCR data.

Modelling of PMR – VAC069A

Analysis of VAC069A was performed as for VAC068 but additionally any data point that was negative but preceded a positive data point was replaced with a value = 5 gc/mL; otherwise negative data points occurring after any positive data point but not preceding a positive data point were treated as 0 gc/mL. The time interval between the morning and evening bleeds used for qPCR monitoring in this study was set as 0.37 days. PMR per 48 hours was then calculated using a linear model fitted to $\log_{10}$-transformed qPCR data.

Peripheral blood mononuclear cell (PBMC), plasma and serum preparation

Blood samples were collected into lithium heparin-treated vacutainer blood collection systems (Becton Dickinson, UK). PBMC were frozen in foetal calf serum (FCS) containing 10% dimethyl sulfoxide (DMSO) and stored in liquid nitrogen. Plasma samples were stored at -80 °C. For serum preparation, untreated blood samples were stored at room temperature (RT) and then the clotted blood was centrifuged for 5 min (1000 *xg*). Serum was stored at -80 °C.

Anti-PvDBP_RII standardized ELISA

ELISAs to quantify circulating PvDBP_RII-specific total IgG responses were performed using standardized methodology, similar to that previously described (20). Day C-1 and dC+90 serum or plasma samples from the VAC068 and VAC069A volunteers were tested, alongside samples from 8 healthy UK adults previously vaccinated in the VAC051 Phase Ia trial of a candidate PvDBP_RII vaccine (Group 2C) (20). Nunc MaxiSorp ELISA plates (Thermo Fisher) were coated overnight (≥16 h) at 4 °C with 50 µL per well of 2 µg/mL PvDBP_RII (SalI allele) protein (20). Plates were washed 6x with 0.05 % PBS/Tween (PBS/T) and tapped dry. Plates were blocked for 1 h with 100 µL per well of Starting Block™ T20 (Thermo Fisher) at 20 °C. Test samples were diluted in blocking buffer (minimum dilution of 1:100), and 50 µL per well was added to the plate in triplicate. Reference serum (made from a pool of high-titer vaccinated donor serum) was diluted in blocking buffer in a three-fold dilution series to form a ten-point standard curve. Three independent dilutions of the reference serum were made to serve as internal controls. The standard curve and internal controls were added to the plate at 50 µL per well in duplicate. Plates were incubated for 2 h at 20 °C and then washed 6x with PBS/T and tapped dry. Goat anti-human IgG–alkaline phosphatase secondary antibody (Merck) was diluted 1:1000 in blocking buffer and 50 µL per well was added. Plates were incubated for 1 h at 20 °C. Plates were washed 6x with PBS/T and tapped dry. 100 µL per well of PNPP alkaline phosphatase substrate (Thermo Fisher) was added, and plates were incubated for approximately 15 min at 20 °C. Optical density at 405 nm (OD_{405}) was measured using an ELx808

absorbance reader (BioTek) until the internal control reached an OD_{405} of 1.0. The reciprocal of the internal control dilution giving an OD_{405} of 1.0 was used to assign an arbitrary unit (AU) value of the standard. Gen5 ELISA software v3.04 (BioTek) was used to convert the OD_{405} of test samples into AU by interpolating from the linear range of the standard curve fitted to a four-parameter logistic model. Any test samples with an OD_{405} below the linear range of the standard curve at the minimum dilution tested were assigned a minimum AU value of 5.0.

Anti-PvMSP1$_{19}$ and anti-PvCSP ELISA

Anti-PvMSP1$_{19}$-specific total IgG responses were measured in VAC068 and VAC069A volunteer serum and plasma via indirect ELISA (same test samples as for the PvDBP_RII ELISA). Nunc MaxiSorp ELISA plates (Thermo Fisher) were coated with 50 µL per well of 2 µg/mL PvMSP1$_{19}$ protein (kindly provided by Dr Chetan Chitnis, Institut Pasteur, Paris, France (21)) and left overnight (≥16 h) at 4 °C. Plates were washed 6x with 0.05% PBS/T and tapped dry. Plates were blocked for 1 h with 100 µL per well Starting Block™ T20 (Thermo Fisher) at 20 °C. Test samples were diluted 1:100 in blocking buffer and 50 µL per well was added in duplicate. A 1:6400 dilution of a post-CHMI positive control serum sample was also added in duplicate. Plates were incubated for 2 h at 20 °C and then washed 6x with PBS/T and tapped dry. Goat anti-human IgG–alkaline phosphatase secondary antibody (Merck) was diluted 1:1000 in blocking buffer and 50 µL per well was added. Plates were incubated for 1 h at 20 °C, then washed 6x with PBS/T and tapped dry. 100 µL per well of PNPP alkaline phosphatase substrate (Thermo Fisher) was added and plates were incubated for approximately 20 min at 20 °C. OD_{405} was measured using an ELx808 absorbance reader (BioTek) until the positive control reach an OD_{405} of 1.0. Results are plotted as the mean OD_{405} reading for each test sample. Anti-PvCSP responses were measured in VAC068 samples with a very similar method to anti-PvMSP1$_{19}$. Recombinant PvCSP was the full-length protein (VK210 repeats, Belem strain sequence) with a C-terminal four amino acid C-tag, produced by transient transfection in mammalian HEK293 cells and purified by anti-C-tag affinity chromatography.

Illumina sequencing

Blood samples were used from the two volunteers in VAC068 as follows: first volunteer = 1 x 10 mL packed RBC (dC+11), plus 1 x 1.5 mL + 1 x 2 mL packed RBC (dC+14); second volunteer = 1 x 10 mL packed RBC (dC+11), plus 2 x 2.5 mL packed RBC (dC+14). DNA was extracted using the Qiagen blood DNA midi kit and sequenced with Illumina HiSeq X10 with 150 bp paired end reads.

Long read sequencing

Preparation of schizonts: For the preparation of high molecular weight DNA for long-read sequencing blood samples were collected at diagnosis from volunteers enrolled in VAC069A and used to culture schizonts *ex vivo*. This approach was chosen to maximize the quantity of parasite DNA available. Briefly, 20 mL whole blood was depleted of leukocytes using NWF filters. RBC were then washed in McCoy's medium and resuspended at 3 % hematocrit. Parasite growth medium (McCoy's) was supplemented with 20 % AB human serum, 2.4 mg/mL D-glucose, 25 mM HEPES, 0.2 mM hypoxanthine and flasks were gassed with 5% O_2, 5% CO_2 (in N_2) and incubated at 37 °C. The duration of culture was adapted to allow schizont maturation, based on the dominant life cycle stage present at the start of culture, as determined by Giemsa-stained thin and thick blood smears. Twelve hours prior to end of culture, the protease inhibitor E64 was added at a final concentration of 10 µM to prevent schizont rupture. At the end of the culture red cells were lysed and parasites isolated according to our previously published protocol: https://dx.doi.org/10.17504/protocols.io.brgjm3un. In brief, RBC were washed in PBS and lysed in 0.0075 % saponin for 10-15 min on ice; samples were then centrifuged at 2000 x*g* to pellet schizonts, which were snap-frozen on dry ice; and the supernatant was centrifuged at 18,000 x*g* to pellet less mature parasites (rings and trophozoites) – these were also snap frozen on dry ice. In the following steps for DNA extraction, samples containing different parasite stages were pooled to maximize the yield, and samples from all volunteers (except 01-003) were taken forward for sequencing.

High molecular weight DNA extraction: PvW1 parasite pellets were pooled into four groups. Each group was thawed on ice, resuspended in 200 µL cold PBS and extracted using the Qiagen MagAttract® HMW DNA Kit (blood protocol). This yielded a total of 107 ng high molecular weight DNA with an average fragment size of 78 kbp as measured by Femto Pulse system (Agilent).

Shearing and PacBio library construction and sequencing: The pooled high molecular weight DNA (107 ng) was sheared using a Diagenode Megaruptor 3 (speed setting 30) to an average fragment size of 18.2 kbp. SMRTbell® library preparation and clean up were as described in the manufacturer's protocol for low input DNA: https://www.pacb.com/wp-content/uploads/Procedure-Checklist-Preparing-HiFi-Libraries-from-Low-DNA-Input-Using-SMRTbell-Express-Template-Prep-Kit-2.0.pdf. After a 1.8x Ampure PB bead clean-up to remove fragments below 250 bp, 87 ng PvW1 DNA remained for library preparation, which produced 30 ng of SMRTbell® library for sequencing. The SMRT libraries were sequenced on a single Sequel SMRT Cell 1M and yielded 13 Gb sequence (3.4 Gb unique).

Raw sequencing data

Data are included in the study entitled "PvW1 – a new clone of *Plasmodium vivax* with high quality genome assembly ID 6525"; accession number ERP129582.

Illumina:

4472STDY76983 13=1a - ERS6867716

4472STDY76983 14=1b - ERS6867717

4472STDY76983 15=2a - ERS6867718

4472STDY76983 16=2b - ERS6867719

Pacbio:

DN599117N-A1 or 5987STDY8548200 - ERS3947829

Genome assembly and annotation

The genome assembly and annotation for PvW1 are available from the European Nucleotide Archive under project accession PRJEB45464

PacBio subreads from sample DN599117N-A1 (5987STDY8548200) were used for the assembly. Circular consensus sequencing reads (CCS) were generated from the subreads using PacBio SMRTLink (https://www.pacb.com/support/software-downloads/).

Illumina reads: Samples 4472STDY7698313 (volunteer 1a), 4472STDY7698314 (volunteer 1b) and 4472STDY7698315 (volunteer 2a) from STDY4472 were used for assembly polishing. The Illumina reads were processed with CutAdapt 2.7 (22) to remove adapter sequences.

Decontamination of the sequencing data: To identify contaminant species in the sequencing data, BLAST searches of a randomly selected subset of subread sequences were run against the NCBI nt and nr databases (February 2020 versions) (23). The list of detected contaminants was then extended using Diamond 0.9.22 (24) BLASTX of the PacBio subreads and CCS reads against a database that contained 39,920 apicomplexan protein sequences and 190,075 protein sequences from various bacterial and fungal species. Further classification of sequences by species was performed using BLAST of the CCS reads and subreads against a nucleotide database containing the *P. vivax* P01

reference genome (25) (PlasmoDB release 46), GRCh38.p13 human genome assembly and 225 bacterial or fungal sequences. In parallel with this, the same reference nucleotide sequences were also used for competitive mapping of the PacBio and Illumina reads with Minimap2 2.17-r941 (26). Next, a Kraken 2.0.8-beta (27) database was made that contained multiple reference genomes of *Plasmodium*, as well as the genomes of human and other contaminant species that were detected in the previous steps. This database was used to classify the PacBio subreads, CCS reads and Illumina reads. If a PacBio subread was unambiguously detected as belonging to a contaminant species, other subreads that had been produced from the same zero-mode waveguide (ZMW) were also flagged as contaminants. Information from different contaminant detection methods was combined. BLAST against the NCBI nt database (October 2019 version) was run with the PacBio sequences that still remained unclassified after the previous steps. Illumina reads that remained unclassified after running Kraken were removed from the dataset.

In order to verify the effectiveness of Illumina read set decontamination, the Illumina reads were assembled with LightAssembler (initial public release version) (28), and the resulting assembly was checked for contaminants using Diamond BLASTX. The database for Diamond was the same as previously described for the decontamination of PacBio subreads. No contaminant sequences were detected in the Illumina assembly.

Canu assembly of PacBio data: Palindromic CCS were detected using a script from PacBio's GitHub repository (https://github.com/PacificBiosciences/apps-scripts/blob/master/miscUTILS/missing_adaptors.py). The palindromic CCS were removed from the dataset. Next, the decontaminated PacBio subreads and CCS were pooled and assembled with Canu assembler (29) (with default settings of the pacbio-raw mode, and with the genomeSize=29052596 flag). The resulting assembly was deduplicated by merging contigs with unique overlaps using GAP5 v1.2.14-r3753M (30). The assembly was polished using the Arrow algorithm in PacBio gcpp (version 1, https://github.com/PacificBiosciences/gcpp), followed by three iterations of Pilon 1.23 (31). Decontaminated Illumina reads (pooled from three samples) were used as the input for Pilon. The apicoplast and mitochondrion sequences were circularized using Circlator minimus2 (32). Assembly completeness was assessed using BUSCO 3.0.1 (33). The assembly was annotated using the Glasgow server of Companion (34) (http://protozoacompanion.gla.ac.uk/, February 2020 version). The alignment of proteins to the reference genome was enabled in the Companion run and the rest of the settings were left as default.

Mapping of Illumina reads to estimate the coverage of specific genes: Pooled Illumina reads from three samples that had been processed with CutAdapt and decontaminated with Kraken were mapped to the assembly with Minimap2 using the short read mapping mode ("-ax sr").

VIR gene analysis

Analysis of the diversity and relatedness amongst the VIR genes of PvW1 compared to PvP01, PvT01, PvC01 and Sal-1 was carried out as described in (25).

Statistical analysis

Unless otherwise stated, data were analyzed using GraphPad Prism version 9.1.1 for Windows (GraphPad Software Inc.). All tests used were 2-tailed and are described in the text. A value of $P<0.05$ was considered significant.

Study Approvals

The VAC068 and VAC069 trials were registered on ClinicalTrials.gov (NCT03377296 and NCT03797989, respectively) and were conducted according to the principles of the current revision of the Declaration of Helsinki 2008 and in full conformity with the ICH guidelines for Good Clinical Practice (GCP). All volunteers signed written consent forms, and consent was checked to ensure volunteers were willing to proceed prior to CHMI. The VAC068 study received ethical approval from the UK NHS Research Ethics Service (Oxfordshire Research Ethics Committee A, Ref 17/SC/0389). The VAC069 study received ethical approval from the UK NHS Research Ethics Service (South Central – Hampshire A Research Ethics Committee), Ref 18/SC/0577.

GCP compliance for both trials was independently monitored by the University of Oxford Clinical Trials and Research Governance (CTRG) Office. An independent local safety monitor and safety monitoring committee acted as independent experts, who, if required, could evaluate any adverse events and advise the Investigators on treating or referring a volunteer to secondary care.

Supplementary Results

VAC068: Long-term safety monitoring

Follow-up clinic visits at dC+45 and dC+90 gave rise to no safety concerns or indication of relapsing infection, with negative qPCR readings recorded at both time-points. Ongoing annual follow-up by email will continue for 5 years post-CHMI, however, as of time of writing (May 2021; 3 years post-primaquine treatment) no relapse of *P. vivax* has been diagnosed for either volunteer. One volunteer did report symptoms of rapid-onset fever, chills, myalgia and headache, following a prodrome of fatigue and sore throat, 12 months post-CHMI. However, immediate testing for *P. vivax* by qPCR (plus RDT and thick film microscopy) was negative and fevers resolved with no specific treatment within 3 days, with residual symptoms fully resolving within 6 days. The working diagnosis was an intercurrent viral infection (blood tests showed a leukocytosis (11.96 x10^9/mL) with a relative grade 1 neutrophilia (10.7 x10^9/mL) and grade 3 lymphocytopenia (0.47 x10^9/mL); CRP 37 mg/L; urea, creatinine and liver function tests within normal limits). The same volunteer had a similar short-lived febrile illness 20 months and then 22 months post-CHMI but both resolved spontaneously after a few days with no specific treatment and no need for medical attendance.

Supplementary Figures

A

Mosquito Batch Number	Mosquitoes Dissected for Oocyst Count (n)	Mean [range] Oocyst Count on Days 6-7	Mosquitoes Dissected for Spz Count (n)	Median [range] Spz Score in Salivary Glands on Day 14	PvCSP-typing	Genotyping
**C01-001**	5	9 [4-12]	N.D.	N.D.	VK210	Probably mixed-infection
**C05-001**	10	3 [0-6]	10	+2 [0 to +4]	VK210	Mono-infection
**C05-002**	5	19 [15-35]	N.D.	N.D.	VK210	Mixed-infection (2 strains)

B

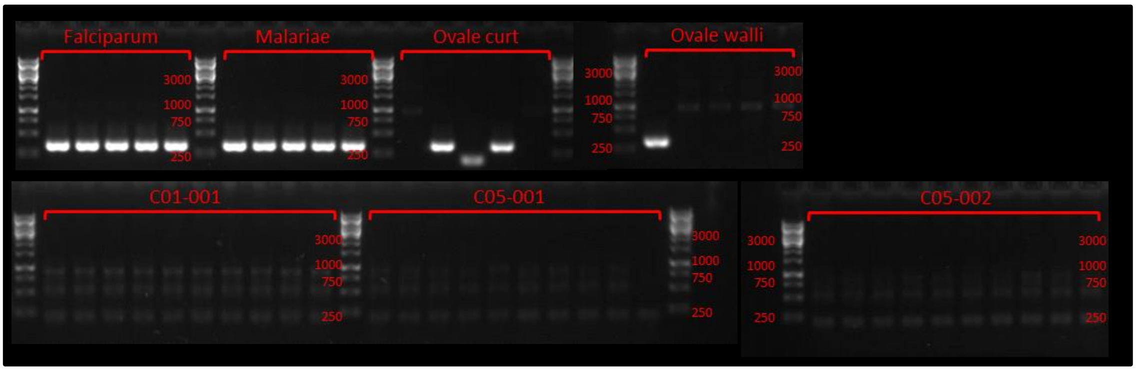

C

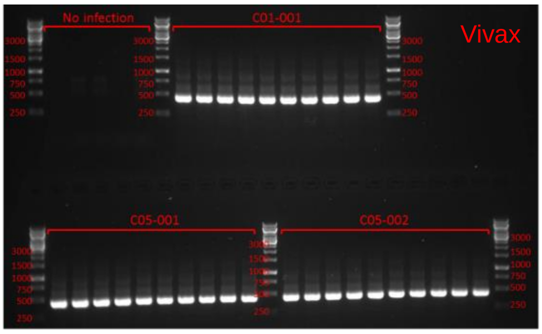

Figure S1. Results of mosquito batch analyses conducted in real-time.

(**A**) Three independent batches of mosquitoes were prepared in Thailand. Oocyst count data from dissected midguts and sporozoite (spz) count data from dissected salivary glands are reported. Score of +2 indicates >10-100 spz observed; score of +4 indicates >500 spz observed; 0 indicates negative; N.D. = not done. Genotyping of patient blood samples was performed to assess multiplicity of *P.*

vivax infection, with extracted DNA processed using a SNP barcode panel as described by the MalariaGEN network (4). Out of the three samples, the barcode sequences for two contained heterozygous genotype calls, suggesting the presence of more than one genotype within the infection. One blood sample (C05-001) had homozygous calls at all single nucleotide polymorphisms (SNPs), suggesting that it contained only a single *P. vivax* genotype. (**B**) Nested PCR to test for presence of *P. falciparum*, *P. malariae*, *P. ovale curtisi* and *P. ovale wallikeri* DNA. Amplified band at ~300 bp shown presence of DNA from each species of *Plasmodium* parasite. Positive control samples were tested in five replicates, with successful amplification in 5/5 cases for *P. falciparum* and *P. malariae*, but less reliable amplification for the two subspecies of *P. ovale*. Each of the three donor blood samples used to produce the batches of mosquitoes were tested in ten replicates. All test samples were negative. (**C**) The same PCR assay was performed using primers for *P. vivax*. All ten replicates for each donor blood sample were positive. Non-infected blood sample control was negative (tested in five replicates).

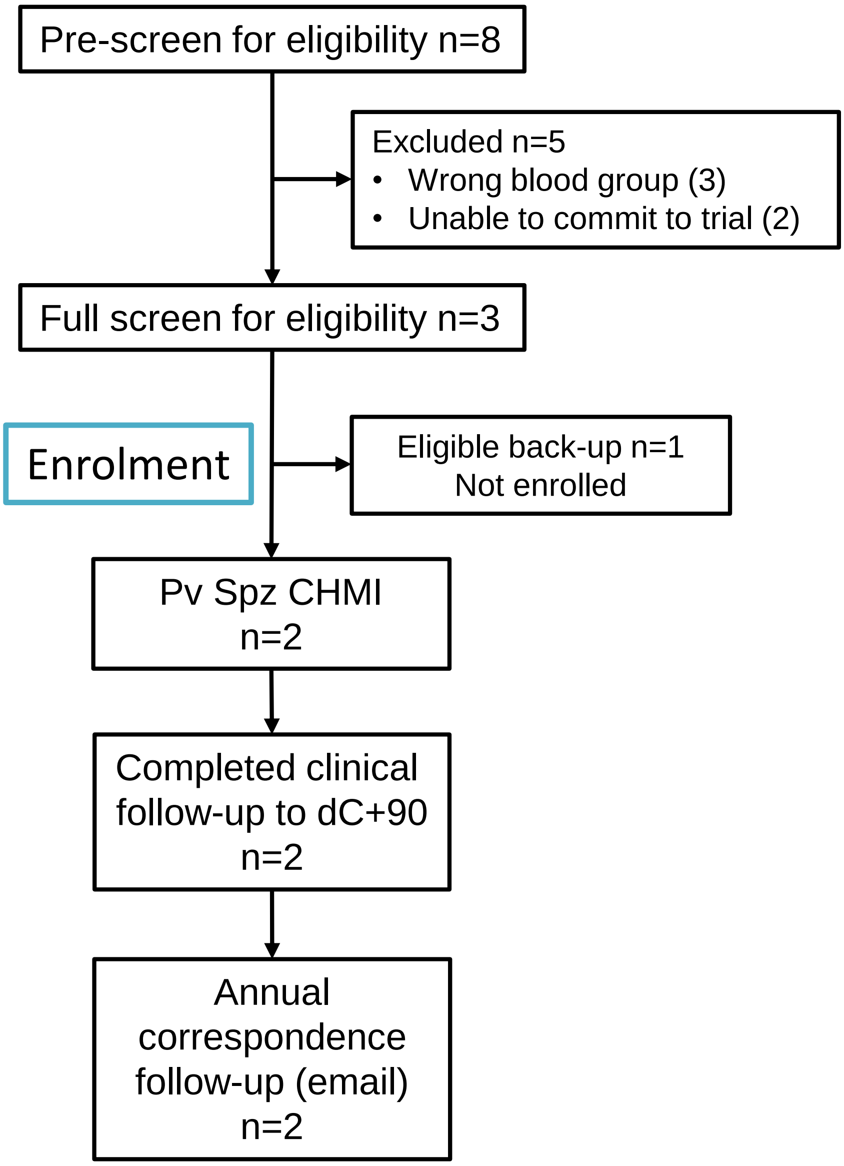

Figure S2. VAC068 flow chart of study design and volunteer recruitment.

Enrolment into the VAC068 study began in April 2018. Eight volunteers underwent pre-screening for O- blood group prior to full screening. Three eligible volunteers were identified, of whom one was a back-up (in case a volunteer withdrew prior to CHMI) and two proceeded to enrolment and underwent *P. vivax* CHMI delivered by mosquito-bite on 13 April 2018. Clinical follow-up continued until 90 days after challenge (dC+90) and was completed by 16 July 2019. Annual follow-up by email correspondence will continue for 5 years. Volunteer demographics are summarized in **Table S1**.

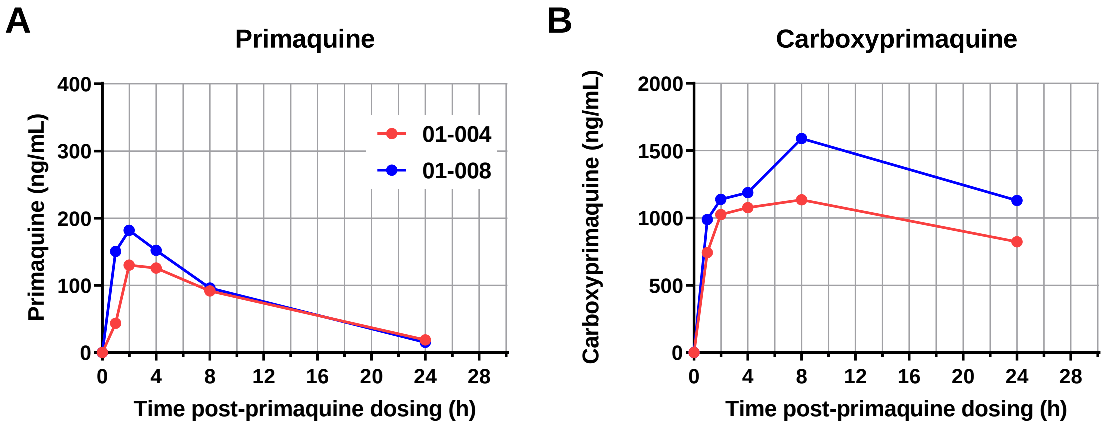

C

Primaquine kinetic parameter	Vol ID: 01-004	Vol ID: 01-008
C_{max} (ng/mL)	130.3	181.8
t_{max} (h)	2	2
AUC_{inf} (h*ng/mL)†	1,878	2,094
$t^{1/2}$ (h)†	7.2	6

Figure S3. Results of primaquine test dose at screening for the VAC068 volunteers.

Plasma concentrations of (**A**) primaquine (parent drug) and (**B**) carboxyprimaquine (metabolite) versus time in the two VAC068 volunteers following a single 30 mg oral dose of primaquine. Results for carboxyprimaquine, the major observed plasma metabolite of primaquine, are shown for completeness, but this is not produced primarily via CYP2D6 metabolism (10) and is, therefore, an unproven indicator of CYP2D6 phenotype and primaquine efficacy. (**C**) Pharmacokinetic parameters of primaquine. † Data previously reported by Bennett *et al.* (9) indicated that these kinetic parameters were significantly different between subjects that relapsed with *P. vivax* versus those that did not, consistent with decreased metabolism of primaquine. The primaquine datasets for both volunteers in VAC068 are consistent with the expected ranges for CYP2D6 extensive metabolizers as reported by Bennett *et al.* (9).

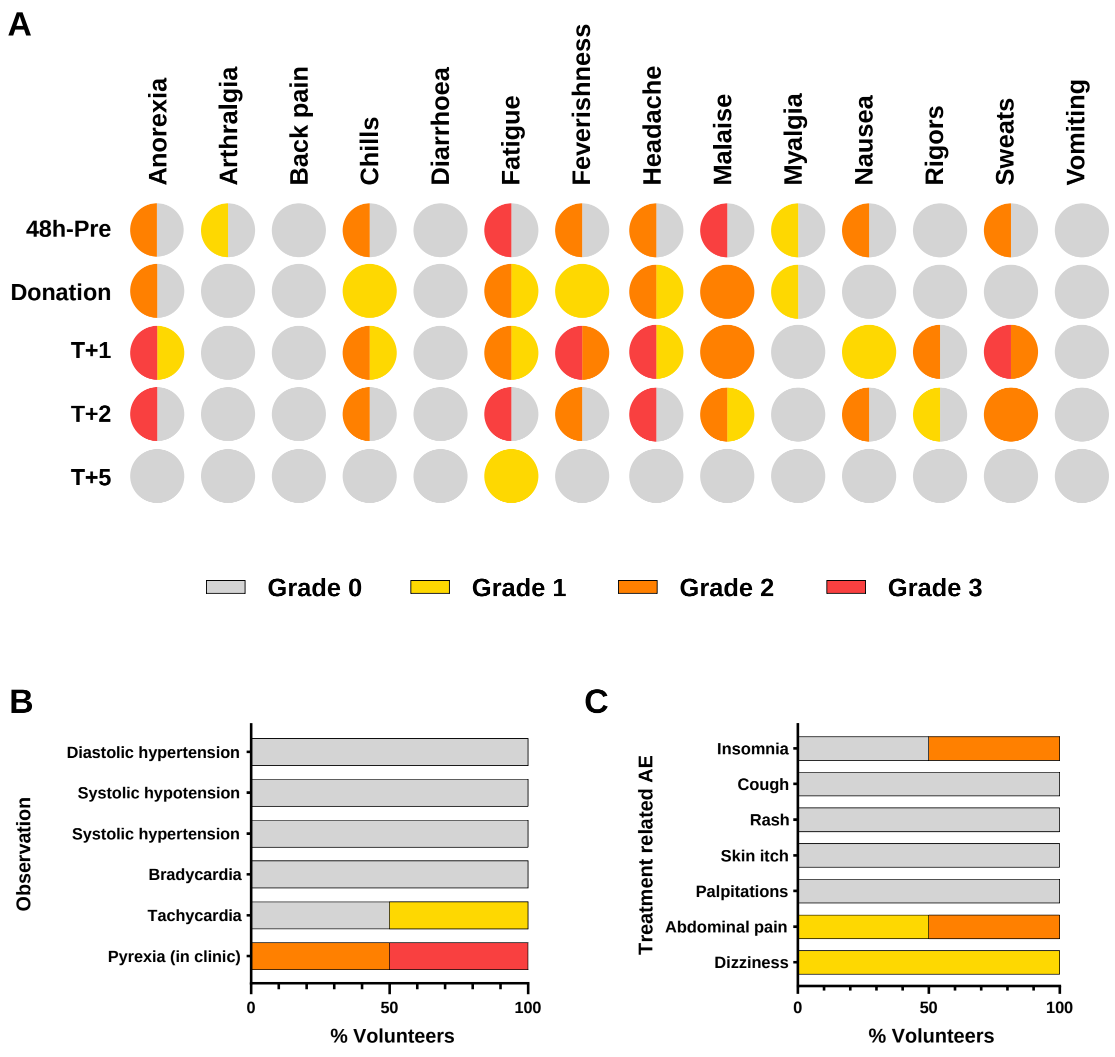

Figure S4. VAC068 adverse event data following *P. vivax* sporozoite CHMI.

(**A**) The solicited systemic AEs recorded at the indicated time-points during the CHMI period are shown as the maximum severity reported by each volunteer and as a percentage of the volunteers reporting each individual AE (n=2). Color-coding refers to AE grading: 0 = none; 1 = mild; 2 = moderate; 3 = severe. 48h-pre = the 48 hour period prior to blood donation; Donation = time-point of blood donation; +1, +2 and +5 days post-treatment (T). (**B**) Percentage of all volunteers (n=2) with AEs in recorded physical observations during the CHMI period (from day of challenge up until 90 days post-challenge). Pyrexia relates to the measured temperature during clinic visits. (**C**) AEs possibly relating to anti-malarial treatment (from day T+1 until completion of primaquine treatment course on T+16) shown as a percentage of all volunteers (n=2).

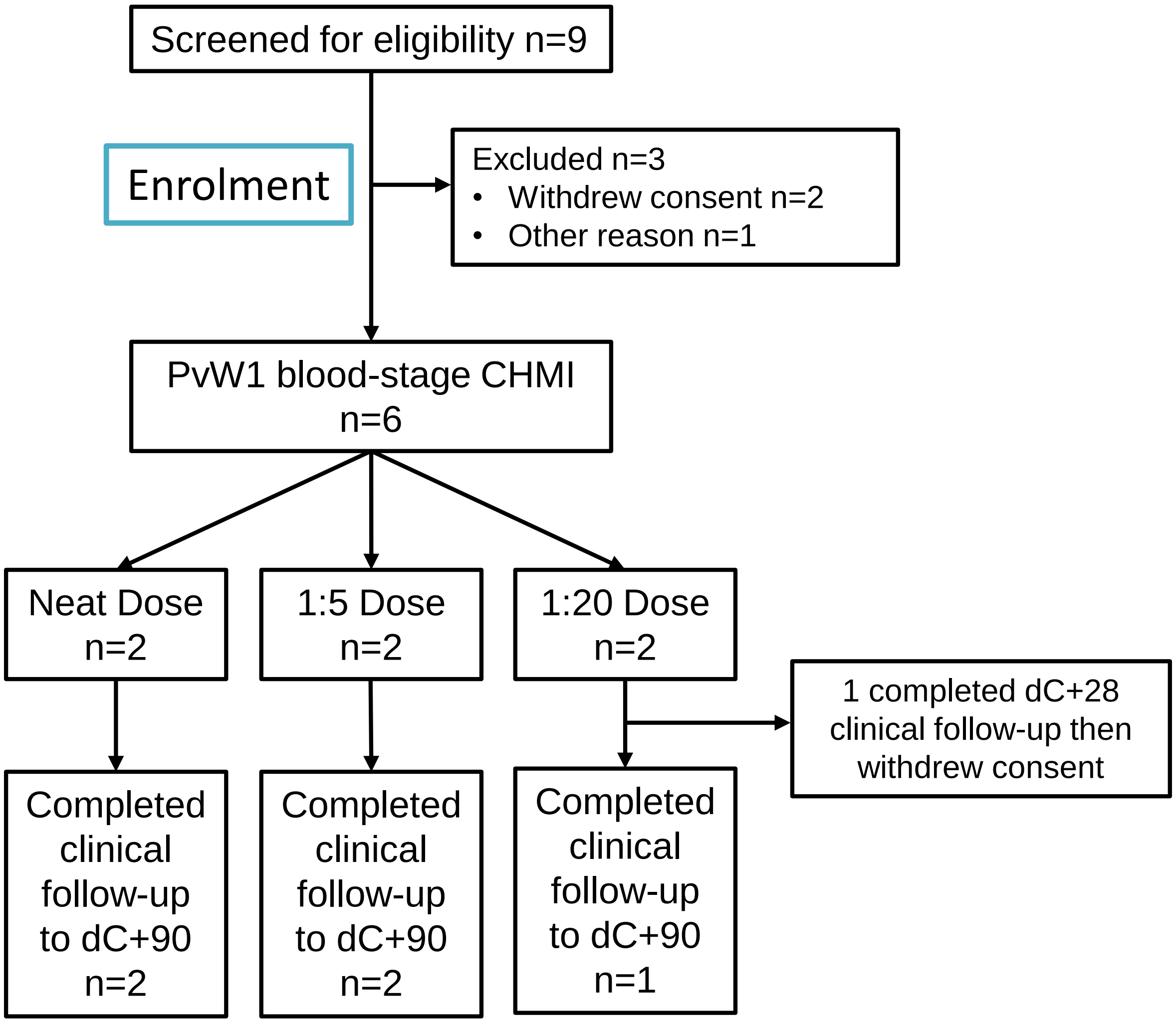

Figure S5. VAC069A flow chart of study design and volunteer recruitment.

Enrolment into the VAC069A study began in January 2019. Six volunteers (of the nine screened) were enrolled into the blood-stage CHMI trial – two into each dose group (neat, 1:5, 1:20 dilution). One volunteer in the 1:20 dose group completed the C+28 follow-up but then withdrew consent. The five remaining volunteers completed their follow-up until C+90. Across the groups, 4 males and 2 females were enrolled, the mean age was 27 years (range 18 – 33 years). None had previously travelled to malaria-endemic regions.

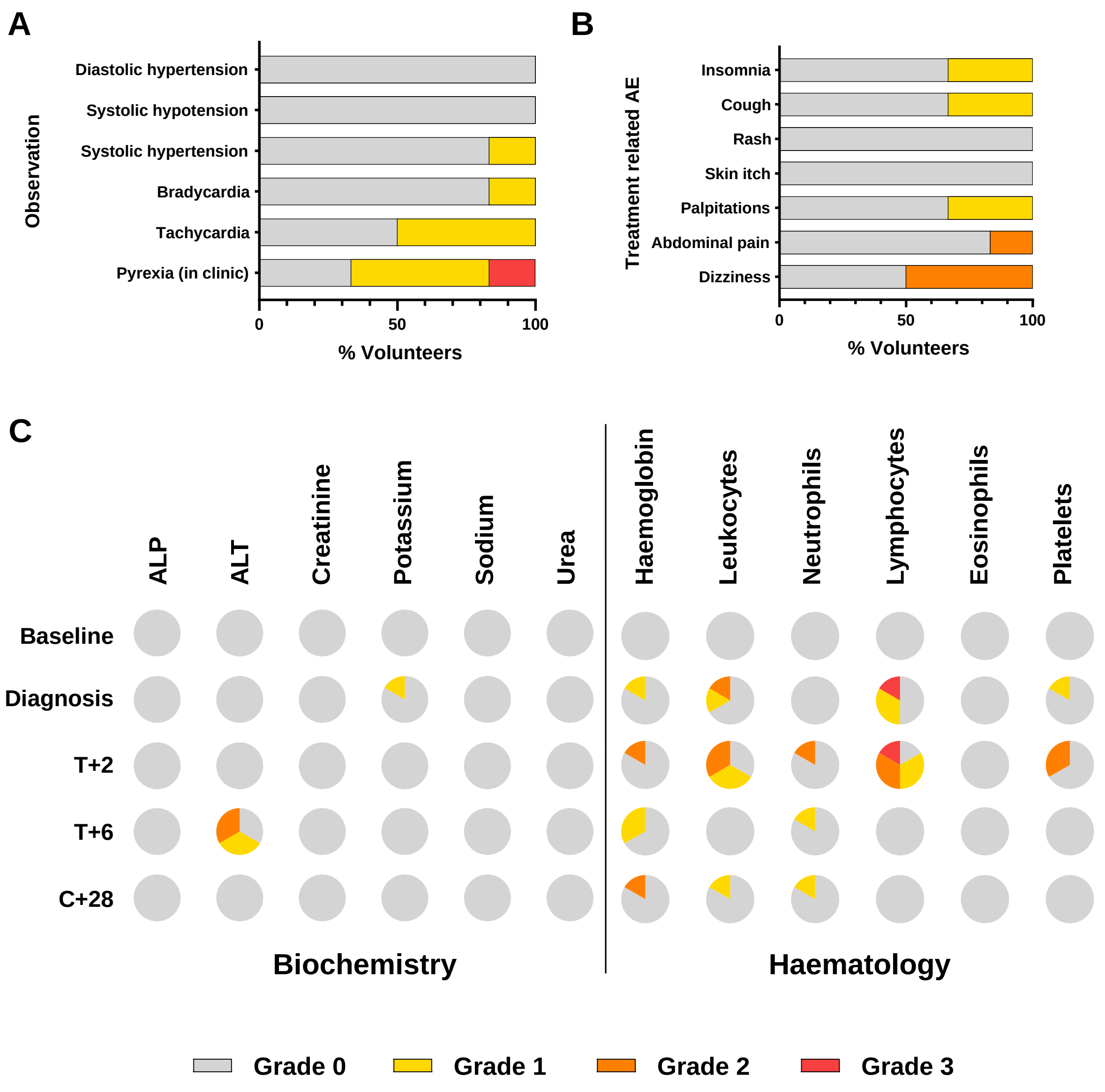

Figure S6. VAC069A adverse event data following *P. vivax* PvW1 blood-stage CHMI.

(**A**) Percentage of all volunteers (n=6) with AEs in recorded physical observations (from day of challenge up until 90 days post-challenge). Pyrexia relates to the measured temperature during clinic visits. (**B**) AEs possibly relating to anti-malarial treatment, recorded from day after starting treatment (T+1) until 28 days post-challenge (C+28, approximately 10 days post-completion of treatment), shown as a percentage of all volunteers (n=6). Color-coding refers to AE grading: 0 = none; 1 = mild; 2 = moderate; 3 = severe. **(C)** The laboratory AEs recorded at the indicated time-points during the CHMI period (day of challenge until 90 days post-challenge) are shown as the maximum severity

reported for each volunteer and as a percentage of the volunteers reporting each individual AE (n=6). Baseline = prior to CHMI (C-1); Diagnosis = time-point of malaria diagnosis; +2 and +6 days post-treatment (T+2, T+6); C+28 = 28 days post-CHMI.

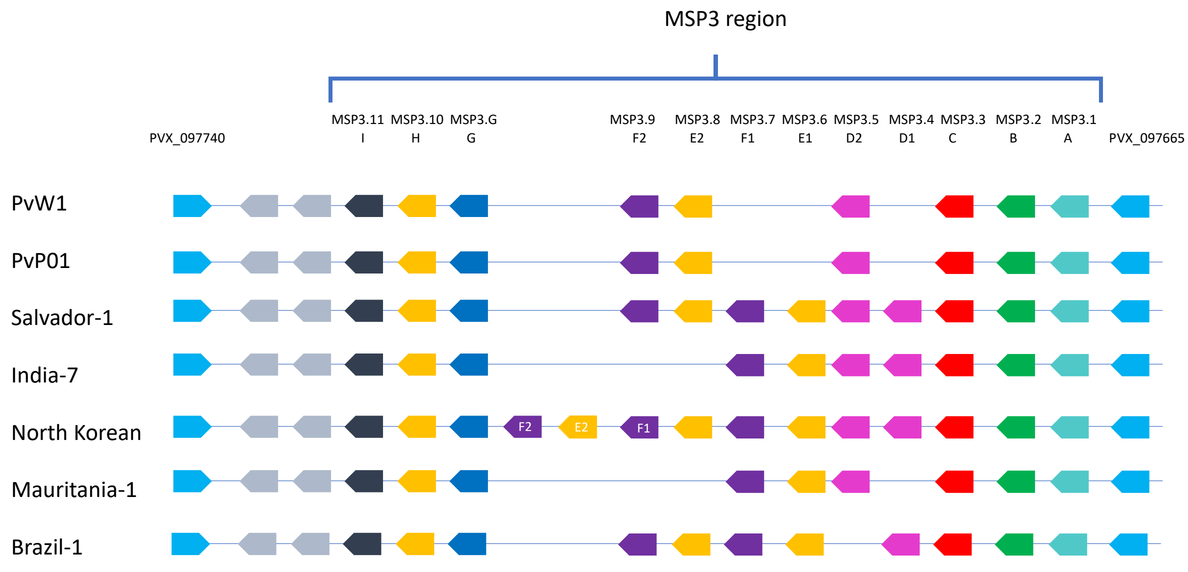

Figure S7. Organization of the MSP3 multigene family in PvW1 compared to other *P. vivax* isolates.

Organization of the MSP3 multigene family in PvW1 is compared to *P. vivax* isolates: PvP01 (25) and Salvador-1, India-7, North Korean, Mauritania-1, Brazil-1 (35). Flanking genes are shown and are syntenic across all isolates, as are MSP3.1, MSP3.2, MSP3.3, MSP3.G, MSP3.10 and MSP3.11. There is variability in the central region of the MSP3 region in the presence or absence and copy number of MSP3.4, MSP3.5, MSP3.6, MSP3.7, MSP3.8 and MSP3.9.

A PvCSP

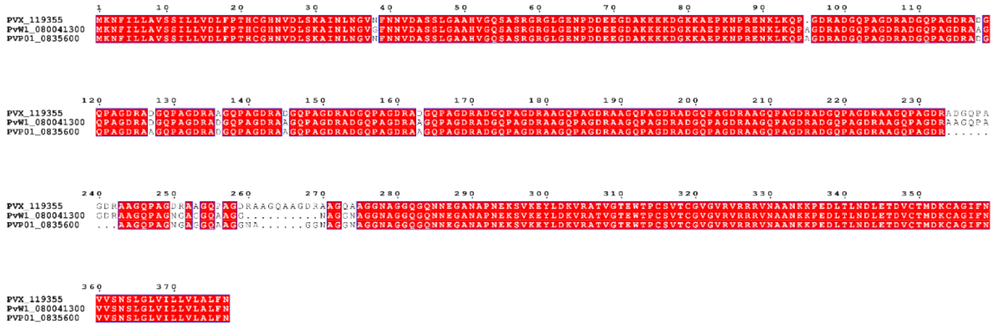

B Pvs25

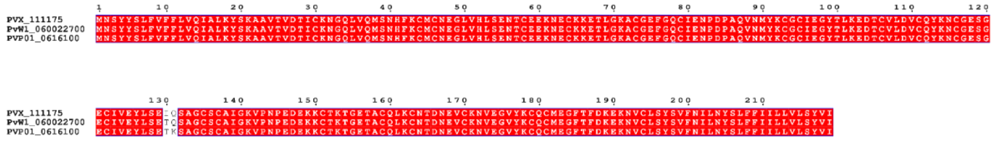

C PvDBP

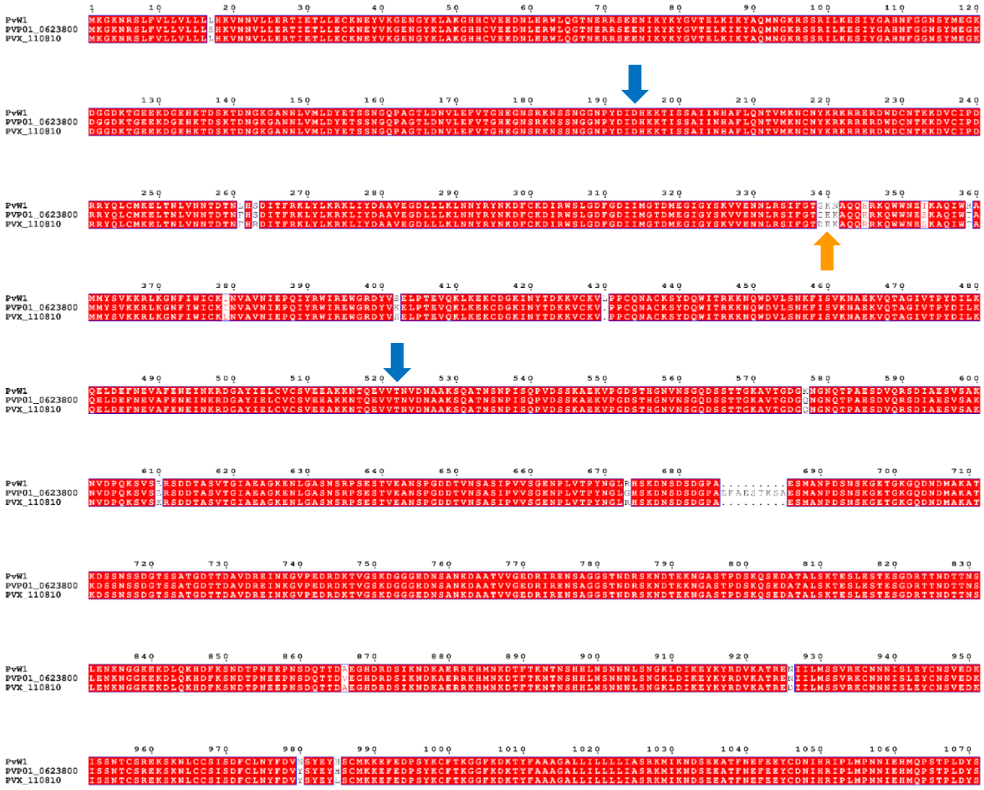

Figure S8. Amino acid sequence alignments of leading *P. vivax* vaccine candidate antigens.

Sequences are aligned for (**A**) sporozoite-stage PvCSP, (**B**) transmission-stage Pvs25, and (**C**) blood-stage PvDBP from PvW1, PvP01 and SalI (labelled PVX). The start and end of region II in PvDBP, present in two clinical vaccine candidates (20, 36), are indicated by the blue arrows (D194-T521, numbered as in SalI). The "DEK epitope" (37) is indicated by the orange arrow.

A

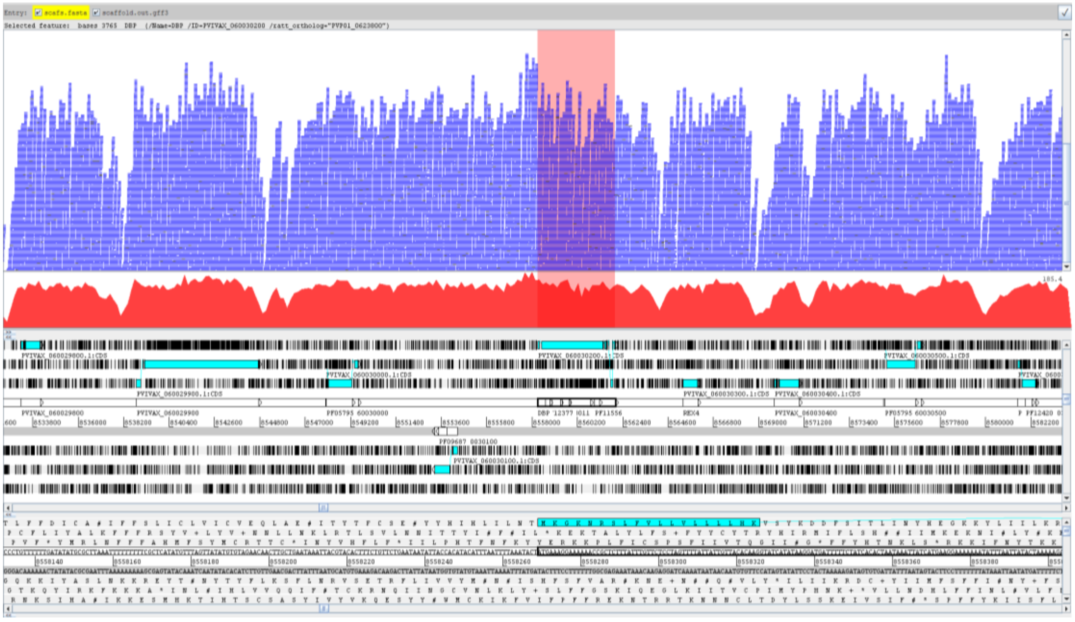

B

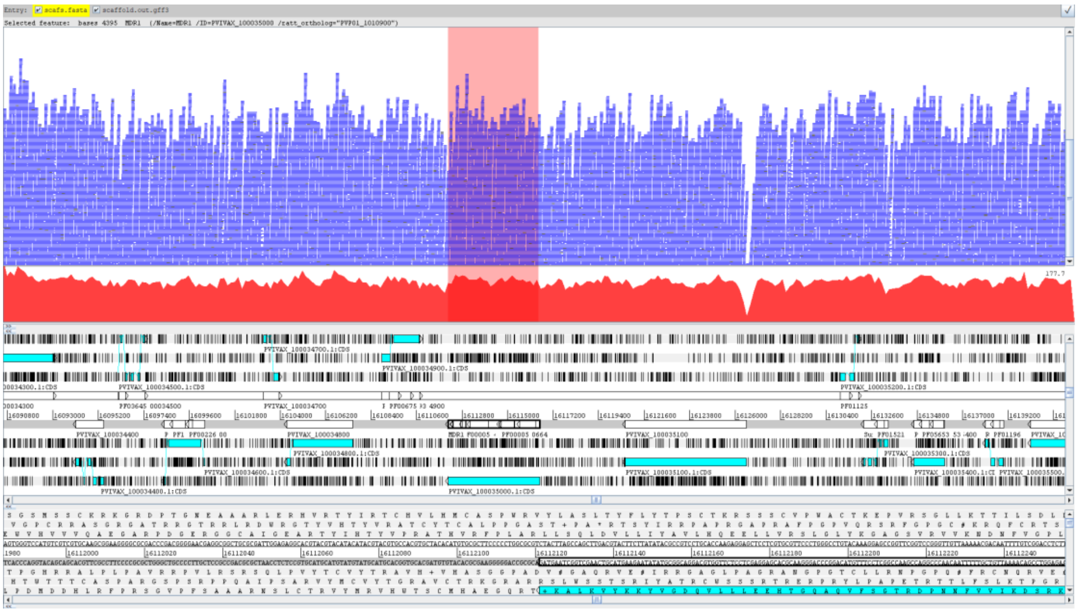

C

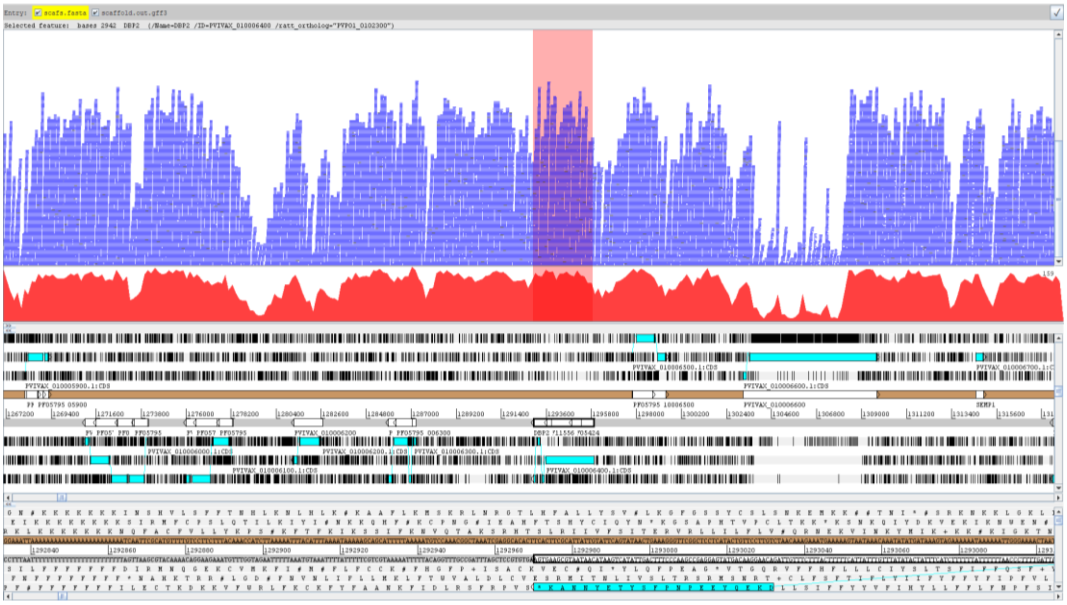

D

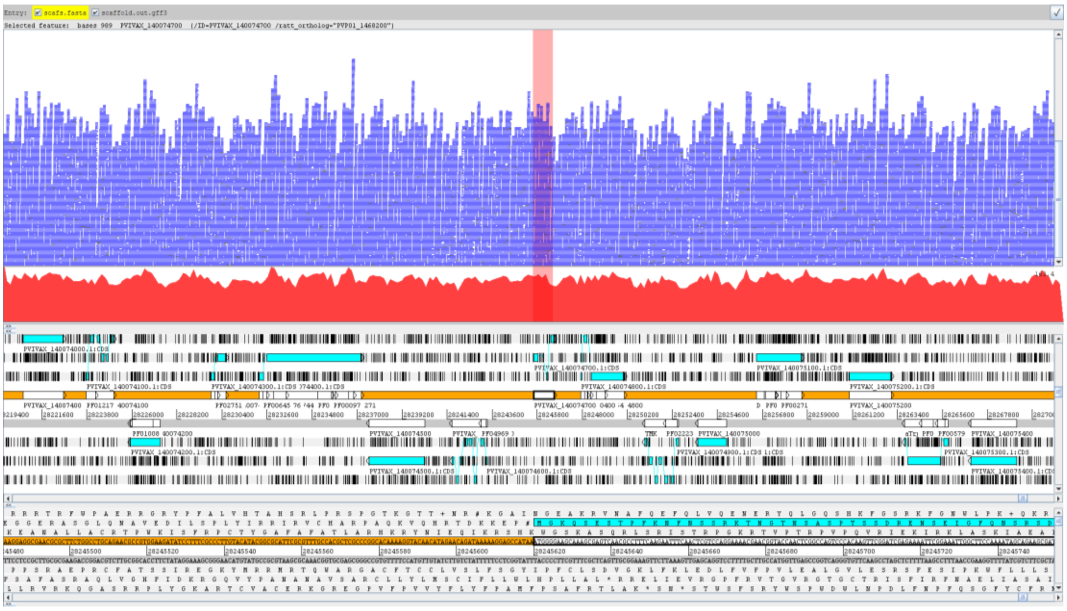

Figure S9. Analysis of gene copy number variation in PvW1.

Illumina reads were mapped to the new PvW1 genome assembly to determine the copy number of genes known to be present in multiple copies in some *P. vivax* isolates. Regions analyzed were (**A**) PvDBP; (**B**) PvMDR1; (**C**) PvDBP2; and (**D**) PvP01_1468200. Individual reads (blue) and coverage (red) are mapped above the genome assembly. The individual genes of interest are highlighted in pink. In all cases Illumina read coverage over the gene of interest appears to be consistent with the flanking

regions suggesting that these genes are present in single copy in the PvW1 genome. Note that the genes labelled PVIVAX are in fact PvW1.

Supplementary Tables

Demographic	Volunteer 1	Volunteer 2
**Sex**	Female	Male
**Age (5-year range)**	30-35	20-25
**BMI**	19.8	23.2
**Ethnicity**	Caucasian	Caucasian
**Blood group (ABO and rhesus)**	O negative	O negative
**Duffy blood group sero-phenotype**	Fya+Fyb+	Fya+Fyb+
**G6PD (U/gHb)**[a]	9.0	7.7
**HLA-type**	Typing Done	Typing Done
**Hemoglobinopathy screen**	Negative	Negative
**CMV sero-status**	Positive	Positive
**EBV sero-status**	Positive	Positive
**CYP2D6 genotype (predicted phenotype**[b]**)**	CYP2D6*1/*1 (EXT)	CYP2D6*1/*4 (EXT)
**CYP2D6 metabolizer phenotype predicted by Activity Score (AS)-Model A**[c]	Score 2 (EXT)	Score 1 (EXT)

Table S1. Demographic information for the two volunteers in the VAC068 study.

[a] Normal G6PD range = 5.8 – 18.8 U/gHb.

[b] Predicted CYP2D6 metabolizer phenotype according to (8) by service provider.

[c] Predicted CYP2D6 metabolizer phenotype according to (38), as also used in the study by Bennett *et al*. (9). AS-Model A scores range from 0 to 2, with 0 indicating poor or no CYP2D6 activity, 0.5 intermediate, and 1 or 2 indicating normal levels of CYP2D6 activity.

EXT = extensive metabolizer.

Volunteer	DoD	D6.5	D7	D7.5	D8	D8.5	D9	D9.5	D10
01-004	14.5	N	N	N	8	54	80	34	181
01-008	14	N	N	N	N	79	119	119	155

Volunteer	DoD	D10.5	D11	D11.5	D12	D12.5	D13	D13.5	D14
01-004	14.5	302	666	1036	1468	2517	8466	11449	18076
01-008	14	394	1026	1717	1865	2889	8841	16388	16717

Volunteer	DoD	D14.5	T+1	T+2	T+4	T+10	T+16	C+45	C+90
01-004	14.5	31010	610	0.2	1.1	N	N	N	0.4
01-008	14		52	0.9	N	N	N	N	0.0

Table S2: Raw qPCR data (genome copies/mL) for VAC068.

Top row represents day of follow-up visit post mosquito-bite CHMI. Data highlighted in red represent qPCR measurement at time-point of blood donation, also referred to as “day of donation” (DoD) for a particular individual. N = PCR negative for all three triplicate readings in the assay. Squares highlighted in grey indicate negative or a mean reading <20 gc/mL (below minimum positive reporting criteria). Squares highlighted in blue indicate samples taken post-treatment: T+1 indicates 1 day immediately post-treatment initiation; longer-term clinical follow-up occurred at days C+45 and C+90.

A

MEDDRA System Organ Class	MEDDRA Higher Level Term	MEDDRA Preferred Term	Subject ID (diagnosis day)	Onset (days post-CHMI)	AE duration (days)	Maximum severity
Gastrointestinal disorders	Gastrointestinal and abdominal pains (excl oral and throat)	Abdominal pain upper	01-004	11	2	Grade 1
Musculoskeletal and connective tissue disorders	Joint related signs and symptoms	Arthralgia	01-004	12	2	Grade 1
Musculoskeletal and connective tissue disorders	Musculoskeletal and connective tissue pain and discomfort	Back pain	01-004	13	1	Grade 1
Cardiac disorders	Cardiac signs and symptoms NEC	Dizziness	01-008	11	1	Grade 1

B

MEDDRA System Organ Class	MEDDRA Higher Level Term	MEDDRA Preferred Term	Subject ID	Onset (days post-CHMI)	AE duration (days)	Maximum severity
Nervous system disorders	Migraine headaches	Migraine	01-004	66	2	Grade 3

C

Laboratory AE	Volunteer	Time-point	Result	Grade	Action taken
Hyber-bilirubinaemia (µmol/L)	01-008	C-1	32	2	No clinical concern, normal ALT/ALP. Resolved by C+14 (20 µmol/L). No action taken.
Anaemia (g/L)	01-008	C+47	123	1	No clinical concern, resolved by C+94 (13.1 g/L). No action taken.
Low white blood cell count ($x10^9$/L)	01-004	C+14	3.03	1	Consistent with *P. vivax* diagnosis, repeated and resolved by C+46 (5.77 $x10^9$/L).
Lymphocyto-paenia ($x10^9$/L)	01-008	C+14	0.67	2	Consistent with *P. vivax* diagnosis, repeated and resolved by C+47 (1.95 $x10^9$/L).
	01-004	C+14	0.56	2	Consistent with *P. vivax* diagnosis, repeated and resolved by C+47 (1.89 $x10^9$/L).

Table S3: VAC068 unsolicited, grade 3 and laboratory AEs.

(**A**) Unsolicited AEs deemed at least possibly (possibly, probably or definitely) related to *P. vivax* sporozoite CHMI in all volunteers (n=2) are shown, with MedDRA coding and maximum severity

reported. (**B**) Onset and duration of grade 3 AEs: only one grade 3 unsolicited AE (not related to CHMI) was reported by volunteer 01-004. Headache, starting at C+66, lasted 2 days and involved attendance to the volunteer's medical practitioner, but resolved fully. The reported headache was very similar to the volunteer's previous migraines. Grade 3 solicited AEs are shown in **Fig. 1B** and **S4**. (**C**) Laboratory AEs in all volunteers (n=2) in the VAC068 study, from day of challenge until 90 days post-challenge. Blood was drawn for haematology and biochemistry pre-CHMI (day C-1) and post-CHMI on days C+9, C+11, on day of blood donation (C+14) with an additional draw for haematology within 12 hours of treatment, and finally at C+45 and C+90. Blood tests were also carried out at other time-points if clinically indicated. Maximum reported severity is shown, graded as per site-specific grading tables; (Grading: 1 = mild; 2 = moderate; 3 = severe). ALT = alanine aminotransferase; ALP = alkaline phosphatase.

Volunteer	DoD	D0	D1	D2	D2.5	D3	D3.5	D4	D4.5	D5	D5.5	D6	D6.5
01-002	15.5	N	N	N	N	N	N	N	N	N	20	N	7
01-003	12.5	N	N	N	N	N	N	N	N	11	6	11	63
01-005	15	N	N	N	N	N	N	N	N	N	N	N	N
01-006	15	N	N	N	N	N	N	N	N	N	N	N	N
01-007	15.5	N	N	N	N	N	N	N	N	N	N	N	N
01-009	16.5	N	N	N	N	N	N	N	N	N	N	2	15

Volunteer	DoD	D7	D7.5	D8	D8.5	D9	D9.5	D10	D10.5	D11	D11.5	D12	D12.5
01-002	15.5	17	6	17	51	58	95	67	156	301	290	294	411
01-003	12.5	78	45	60	260	508	407	1060	2060	3132	2640	5393	11259
01-005	15	N	5	18	22	72	44	239	250	519	611	972	1735
01-006	15	6	N	21	21	33	38	8	227	266	317	459	986
01-007	15.5	N	N	N	23	32	58	57	204	406	207	616	1227
01-009	16.5	N	N	N	13	15	16	8	55	53	24	76	305

Volunteer	DoD	D13	D13.5	D14	D14.5	D15	D15.5	D16	D16.5	D17	D17.5	D18	D19
01-002	15.5	956		2251	417	5035	3779		182		168		
01-003	12.5		17		N							N	
01-005	15	2666	2999	4700	1538	17795	14843		13925		1324	246	
01-006	15	1331		2632	1056	8687	7112		294		154		
01-007	15.5	1431	1253	3054	1444	9597	8134	15768		555		355	
01-009	16.5	239		717		1479	1438	2593	9668	7894		534	464

Table S4: Raw qPCR data (genome copies/mL) for VAC069A.

Top row represents day of follow-up visit post blood-stage CHMI. Data highlighted in red represent qPCR measurement at time-point of diagnosis according to protocol, also referred to as "day of diagnosis" (DoD) for a particular individual. N = PCR negative for all three triplicate readings in the assay. Squares highlighted in grey indicate negative or a mean reading <20 gc/mL (below minimum positive reporting criteria). Squares highlighted in orange indicate a sample taken immediately pre-treatment at the next clinic visit following diagnosis; where this occurs, volunteers were officially diagnosed based on symptoms and/or qPCR and/or thick film microscopy data obtained in real time between clinic visits. Squares highlighted in blue indicate samples taken post-treatment.

A

Volunteer	Grade 3 AE	Time-point	Persisted at Grade 3	Resolution
01-002	Feverishness	T+1	24 hours	T+2
01-003	Feverishness	Diagnosis/C+12.5	48 hours	T+2
	Chills	T+1	24 hours	T+2
	Sweats	T+1	24 hours	T+2
	Myalgia	T+1	24 hours	T+2
	Fatigue	T+1	24 hours	C+28
	Malaise	T+1	24 hours	T+2
01-006	Feverishness	T+1	24 hours	T+2
	Chills	T+1	24 hours	T+2
	Headache	T+1	24 hours	T+2
	Fatigue	T+1	24 hours	T+2
	Malaise	T+1	24 hours	T+2
01-009	Feverishness	T+1	24 hours	T+2
	Rigor	T+1	24 hours	T+2

B

MEDDRA System Organ Class	MEDDRA Higher Level Term	MEDDRA Preferred Term	Subject ID (diagnosis day)	Onset (days post-CHMI)	AE duration (days)	Maximum severity
Skin and subcutaneous tissue disorders	Pruritus NEC	Pruritus	01-003 (C+12.5)	0	1	Grade 1
Metabolism and nutrition disorders	Appetite disorders	Decreased appetite	01-003 (C+12.5)	11	4	Grade 2
Musculoskeletal and connective tissue disorders	Muscle pains	Myalgia	01-006 (C+15)	11	1	Grade 1

C

Laboratory AE	Volunteer	Time-point	Result	Grade	Action taken
Anaemia (g/L)	01-002	T+6	119	1	Repeated and resolved at next visit (C+28)
	01-005	C+16, C+34 (C+14-C+90)	98 (98-109)	(1-)2	Monitored from C+14 to C+90, AE ongoing at C+90 (102 g/L; Grade 1) Referred to General Practitioner for ongoing medical care, with follow-up of AE ongoing
Low white blood cell count ($x10^9/L$)	01-002	T+1	3.3	1	Consistent with *P. vivax* diagnosis, repeated and resolved (6.53 $x10^9/L$) at next visit (T+6)
	01-003	T+1	2.93	1	Consistent with *P. vivax* diagnosis, repeated and resolved (6.86 $x10^9/L$) at next visit (T+6)
	01-005	T+1 (C+7-C+90)	1.68 (1.68-3.39)	(1-)2	Monitored from C+7 to C+90, AE ongoing at C+90 (3.36 $x10^9/L$; Grade1) Referred to General Practitioner for monitoring / ongoing care
	01-006	T+1 (C+14-T+1)	2.36	2	Monitored from C+14 to T+6, resolved at T+6 (4.84 $x10^9/L$); consistent with *P. vivax* diagnosis
Thrombocyto-paenia ($x10^9/L$)	01-003	T+1	114	2	Consistent with *P. vivax* diagnosis; repeated and resolved (233 $x10^9/L$) at next visit (T+6)
	01-005	T+1 (Diagnosis-T+1)	115 (115-127)	(1-)2	Consistent with *P. vivax* diagnosis; repeated and resolved at T+6 (219 $x10^9/L$)
Neutropaenia ($x10^9/L$)	01-005	C+45 (C+14-C+90)	0.76 (0.76-1.46)	(1-)2	Monitored from C+14 to C+90, AE ongoing at C+90 (1.26 $x10^9/L$; Grade 1) Referred to General Practitioner for monitoring / ongoing care
Lymphocyto-paenia ($x10^9/L$)	01-002	T+1 (Diagnosis-T+1)	0.72 (0.72-0.86)	(1-)2	Consistent with *P. vivax* diagnosis, repeated and resolved at T+6 (1.63 $x10^9/L$)
	01-003	T+1	0.77	1	Consistent with *P. vivax* diagnosis, repeated and resolved at T+6 (2.53 $x10^9/L$)
	01-005	Diagnosis (Diagnosis-T+1)	0.44 (0.44-0.63)	(2-)3	Consistent with *P. vivax* diagnosis, repeated and resolved at T+6 (1.63 $x10^9/L$)
	01-006	T+1 (C+14-T+1)	0.3 (0.3-0.83)	(1-)3	Consistent with *P. vivax* diagnosis, repeated and resolved at T+6 (2.07 $x10^9/L$)

D

Laboratory AE	Volunteer	Time-point	Result	Grade	Action taken
Hyponatraemia (mmol/L)	01-003	C+28	134	1	No clinical concern, no action taken
	01-003	C+90	134	1	No clinical concern, no action taken
Hypokalaemia (mmol/L)	01-002	C+90	3.3	1	No clinical concern, no action taken
	01-005	Diagnosis	3.3	1	Repeated within 24 hours at next visit, resolved (3.7 mmol/L)
	01-003	C+90	3.3	1	No clinical concern, no action taken
ALT (IU/L)	01-003	T+6	135	2	Asymptomatic, repeated in 48 hours (80 IU/L; Grade 1) and then resolved at C+28 (21 IU/L)
	01-006	T+6	56	1	Repeated at next visit (C+28) and resolved (29 IU/L)
	01-007	T+6	80	1	Repeated at next visit (C+28) and resolved (31 IU/L)
	01-009	T+6	114	2	Asymptomatic, repeated in 72 hours and resolved (40 IU/L)

Table S5: VAC069A grade 3, unsolicited and laboratory AEs.

(**A**) Onset and duration of grade 3 solicited AEs reported by all volunteers (n=6) in the VAC069A study. (**B**) Unsolicited AEs deemed at least possibly (possibly, probably or definitely) related to *P. vivax* PvW1 blood-stage CHMI in all volunteers (n=6) in the VAC069A study. These were assigned a MedDRA code, with maximum severity reported. (**C**) Laboratory AEs in full blood count in all volunteers (n=6) in the VAC069A study. (**D**) Laboratory AEs in biochemistry profile (urea and electrolytes, liver function tests). ALT = alanine aminotransferase. Maximum reported severity is shown, graded as per site-specific grading tables; (Grading: 1 = mild; 2 = moderate; 3 = severe). In all panels, time-point(s) = days in relation to day of CHMI (C) or treatment (T).

Supplementary References

1. Nanfack Minkeu F, and Vernick KD. A Systematic Review of the Natural Virome of Anopheles Mosquitoes. *Viruses.* 2018;10(5).
2. Bennett JW, Yadava A, Tosh D, Sattabongkot J, Komisar J, Ware LA, et al. Phase 1/2a Trial of Plasmodium vivax Malaria Vaccine Candidate VMP001/AS01B in Malaria-Naive Adults: Safety, Immunogenicity, and Efficacy. *PLoS Negl Trop Dis.* 2016;10(2):e0004423.
3. Miguel-Oteo M, Jiram AI, Ta-Tang TH, Lanza M, Hisam S, and Rubio JM. Nested multiplex PCR for identification and detection of human Plasmodium species including Plasmodium knowlesi. *Asian Pac J Trop Med.* 2017;10(3):299-304.
4. Jacob CG, Thuy-Nhien N, Mayxay M, Maude RJ, Quang HH, Hongvanthong B, et al. Genetic surveillance in the Greater Mekong Subregion and South Asia to support malaria control and elimination. *medRxiv.* 2020:2020.07.23.20159624.
5. Miller LH, Mason SJ, Clyde DF, and McGinniss MH. The resistance factor to Plasmodium vivax in blacks. The Duffy-blood-group genotype, FyFy. *N Engl J Med.* 1976;295(6):302-4.
6. Payne RO, Griffin PM, McCarthy JS, and Draper SJ. Plasmodium vivax Controlled Human Malaria Infection - Progress and Prospects. *Trends Parasitol.* 2017;33(2):141-50.
7. Baird JK. 8-Aminoquinoline Therapy for Latent Malaria. *Clin Microbiol Rev.* 2019;32(4).
8. Rebsamen MC, Desmeules J, Daali Y, Chiappe A, Diemand A, Rey C, et al. The AmpliChip CYP450 test: cytochrome P450 2D6 genotype assessment and phenotype prediction. *Pharmacogenomics J.* 2009;9(1):34-41.
9. Bennett JW, Pybus BS, Yadava A, Tosh D, Sousa JC, McCarthy WF, et al. Primaquine Failure and Cytochrome P-450 2D6 in Plasmodium vivax Malaria. *N Engl J Med.* 2013;369(14):1381-2.
10. Pybus BS, Sousa JC, Jin X, Ferguson JA, Christian RE, Barnhart R, et al. CYP450 phenotyping and accurate mass identification of metabolites of the 8-aminoquinoline, anti-malarial drug primaquine. *Malar J.* 2012;11:259.
11. Joint Ukbts/Hpa Professional Advisory Committee. *Guidelines for the blood transfusion services in the United Kingdom.* 2013.
12. Cervia JS, Wenz B, and Ortolano GA. Leukocyte reduction's role in the attenuation of infection risks among transfusion recipients. *Clin Infect Dis.* 2007;45(8):1008-13.
13. Engwerda CR, Minigo G, Amante FH, and McCarthy JS. Experimentally induced blood stage malaria infection as a tool for clinical research. *Trends Parasitol.* 2012;28(11):515-21.
14. Payne RO, Milne KH, Elias SC, Edwards NJ, Douglas AD, Brown RE, et al. Demonstration of the Blood-Stage Controlled Human Malaria Infection Model to Assess Efficacy of the Plasmodium falciparum AMA1 Vaccine FMP2.1/AS01. *J Infect Dis.* 2016;213(11):1743-51.
15. McCarthy JS, Griffin PM, Sekuloski S, Bright AT, Rockett R, Looke D, et al. Experimentally induced blood-stage Plasmodium vivax infection in healthy volunteers. *J Infect Dis.* 2013;208(10):1688-94.
16. Minassian AM, Silk SE, Barrett JR, Nielsen CM, Miura K, Diouf A, et al. Reduced blood-stage malaria growth and immune correlates in humans following RH5 vaccination. *Med.* 2021;2:1-19.
17. Douglas AD, Edwards NJ, Duncan CJ, Thompson FM, Sheehy SH, O'Hara GA, et al. Comparison of Modeling Methods to Determine Liver-to-blood Inocula and Parasite Multiplication Rates During Controlled Human Malaria Infection. *J Infect Dis.* 2013;208(2):340-5.
18. Duncan CJ, and Draper SJ. Controlled human blood stage malaria infection: current status and potential applications. *Am J Trop Med Hyg.* 2012;86(4):561-5.
19. Hodgson SH, Juma E, Salim A, Magiri C, Kimani D, Njenga D, et al. Evaluating controlled human malaria infection in Kenyan adults with varying degrees of prior exposure to Plasmodium falciparum using sporozoites administered by intramuscular injection. *Front Microbiol.* 2014;5:686.

20. Payne RO, Silk SE, Elias SC, Milne KH, Rawlinson TA, Llewellyn D, et al. Human vaccination against Plasmodium vivax Duffy-binding protein induces strain-transcending antibodies. *JCI Insight.* 2017;2(12):93683.
21. Devi YS, Mukherjee P, Yazdani SS, Shakri AR, Mazumdar S, Pandey S, et al. Immunogenicity of Plasmodium vivax combination subunit vaccine formulated with human compatible adjuvants in mice. *Vaccine.* 2007;25(28):5166-74.
22. Martin M. Cutadapt removes adapter sequences from high-throughput sequencing reads. *2011.* 2011;17(1):3.
23. Sayers EW, Agarwala R, Bolton EE, Brister JR, Canese K, Clark K, et al. Database resources of the National Center for Biotechnology Information. *Nucleic Acids Res.* 2019;47(D1):D23-D8.
24. Buchfink B, Xie C, and Huson DH. Fast and sensitive protein alignment using DIAMOND. *Nature methods.* 2015;12(1):59-60.
25. Auburn S, Bohme U, Steinbiss S, Trimarsanto H, Hostetler J, Sanders M, et al. A new Plasmodium vivax reference sequence with improved assembly of the subtelomeres reveals an abundance of pir genes. *Wellcome open research.* 2016;1:4.
26. Li H. Minimap2: pairwise alignment for nucleotide sequences. *Bioinformatics.* 2018;34(18):3094-100.
27. Wood DE, and Salzberg SL. Kraken: ultrafast metagenomic sequence classification using exact alignments. *Genome Biol.* 2014;15(3):R46.
28. El-Metwally S, Zakaria M, and Hamza T. LightAssembler: fast and memory-efficient assembly algorithm for high-throughput sequencing reads. *Bioinformatics.* 2016;32(21):3215-23.
29. Koren S, Walenz BP, Berlin K, Miller JR, Bergman NH, and Phillippy AM. Canu: scalable and accurate long-read assembly via adaptive k-mer weighting and repeat separation. *Genome Res.* 2017;27(5):722-36.
30. Bonfield JK, and Whitwham A. Gap5--editing the billion fragment sequence assembly. *Bioinformatics.* 2010;26(14):1699-703.
31. Walker BJ, Abeel T, Shea T, Priest M, Abouelliel A, Sakthikumar S, et al. Pilon: an integrated tool for comprehensive microbial variant detection and genome assembly improvement. *PLoS One.* 2014;9(11):e112963.
32. Hunt M, Silva ND, Otto TD, Parkhill J, Keane JA, and Harris SR. Circlator: automated circularization of genome assemblies using long sequencing reads. *Genome Biol.* 2015;16:294.
33. Waterhouse RM, Seppey M, Simao FA, Manni M, Ioannidis P, Klioutchnikov G, et al. BUSCO Applications from Quality Assessments to Gene Prediction and Phylogenomics. *Mol Biol Evol.* 2018;35(3):543-8.
34. Steinbiss S, Silva-Franco F, Brunk B, Foth B, Hertz-Fowler C, Berriman M, et al. Companion: a web server for annotation and analysis of parasite genomes. *Nucleic Acids Res.* 2016;44(W1):W29-34.
35. Rice BL, Acosta MM, Pacheco MA, Carlton JM, Barnwell JW, and Escalante AA. The origin and diversification of the merozoite surface protein 3 (msp3) multi-gene family in Plasmodium vivax and related parasites. *Mol Phylogenet Evol.* 2014;78:172-84.
36. Singh K, Mukherjee P, Shakri AR, Singh A, Pandey G, Bakshi M, et al. Malaria vaccine candidate based on Duffy-binding protein elicits strain transcending functional antibodies in a Phase I trial. *NPJ Vaccines.* 2018;3:48.
37. Chen E, Salinas ND, Ntumngia FB, Adams JH, and Tolia NH. Structural Analysis of the Synthetic Duffy Binding Protein (DBP) Antigen DEKnull Relevant for Plasmodium vivax Malaria Vaccine Design. *PLoS Negl Trop Dis.* 2015;9(3):e0003644.
38. Gaedigk A, Simon SD, Pearce RE, Bradford LD, Kennedy MJ, and Leeder JS. The CYP2D6 activity score: translating genotype information into a qualitative measure of phenotype. *Clin Pharmacol Ther.* 2008;83(2):234-42.
